# Iron-Catalyzed Cross-Coupling Reactions of Alkyl Grignards with Aryl Chlorobenzenesulfonates

**DOI:** 10.3390/molecules26195895

**Published:** 2021-09-29

**Authors:** Elwira Bisz

**Affiliations:** Department of Chemistry, Opole University, 48 Oleska Street, 45-052 Opole, Poland; ebisz@uni.opole.pl; Tel.: +48-77-452-71-60

**Keywords:** iron, cross-coupling, aryl sulfonates, C–O activation, Fe-catalysis, Kumada cross-coupling

## Abstract

Aryl sulfonate esters are versatile synthetic intermediates in organic chemistry as well as attractive architectures due to their bioactive properties. Herein, we report the synthesis of alkyl-substituted benzenesulfonate esters by iron-catalyzed C(sp^2^)–C(sp^3^) cross-coupling of Grignard reagents with aryl chlorides. The method operates using an environmentally benign and sustainable iron catalytic system, employing benign urea ligands. A broad range of chlorobenzenesulfonates as well as challenging alkyl organometallics containing β-hydrogens are compatible with these conditions, affording alkylated products in high to excellent yields. The study reveals that aryl sulfonate esters are the most reactive activating groups for iron-catalyzed alkylative C(sp^2^)–C(sp^3^) cross-coupling of aryl chlorides with Grignard reagents.

## 1. Introduction

Cross-coupling reactions are considered to be a fundamental tool in modern organic synthesis [1,2,3]. In this context, iron catalysis is of great interest in cross-coupling reactions due to the abundance of iron in the earth’s crust, its low toxicity, and easy removal of iron salts from post-reaction mixtures [4,5,6,7,8,9,10,11,12,13]. In addition to the ecological and economic aspects, the great advantage of iron catalysis is also the possibility of using iron in the traditionally challenging C(sp^2^)–C(sp^3^) cross-couplings employing alkyl Grignard reagents possessing β-hydrogens, which are usually difficult because of the propensity of alkyl organometallics to undergo homo-coupling and β-hydride elimination [14,15]. In recent years, iron-catalyzed cross-couplings have attracted significant attention in the pharmaceutical industry [16], where they have become competitive with palladium catalysts, which have historically dominated this field [17,18,19]. However, despite the main thrust towards developing sustainable cross-coupling technologies, the most common ligand in iron catalysis is reprotoxic NMP (NMP = N-methylpyrrolidone), the main role of which is to stabilize the low valent iron species, thus facilitating the coupling [20]. Recent reports presented by us showed that NMP can be successfully replaced with other benign amide-type donors (conjugation of N_lp_ to C=O) [21,22,23,24].

**Figure 1:**
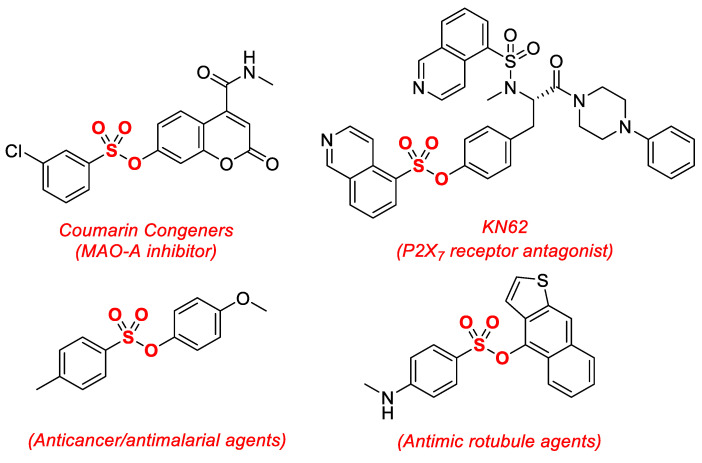
Representative bioactive compounds and pharmaceuticals containing sulfonate ester group.

Aryl sulfonate esters are deemed attractive molecules due to their unique biological properties in medicinal chemistry (Figure 1) [25,26,27,28,29]. Furthermore, aryl sulfonate esters play an important role in organic chemistry as synthetic intermediates, which is related to their versatile application as protecting groups and C–O electrophiles in organic reactions. The resistance of the sulfonate groups to various reaction conditions makes them handy protecting groups for phenols [30,31], while the possibility to activate the C–O bond using transition metal catalysis makes them a valuable alternative to aryl halides in cross-coupling reactions [32,33,34,35,36]. It is worth noting that in both variants, the resulting target products do not contain a sulfonate ester moiety, which is deconstructed under basic or transition-metal-catalyzed conditions. Thus far, attempts have been made to obtain functionalized sulfonate esters only in Sonogashira and Suzuki–Miyaura cross-couplings; however, even these precedents have been limited to few examples of such reactions [37,38,39]. Importantly, both of these methodologies required the presence of expensive and toxic palladium–phosphine catalysts demanding reaction conditions and were limited to C(sp) or C(sp^2^) nucleophiles. Hence, the ability to functionalize aryl sulfonate esters with high cross-coupling selectivity using sustainable iron catalysis while preserving the sulfonate ester moiety would represent an attractive approach to the synthesis of aryl sulfonate esters.

**Figure 2:**
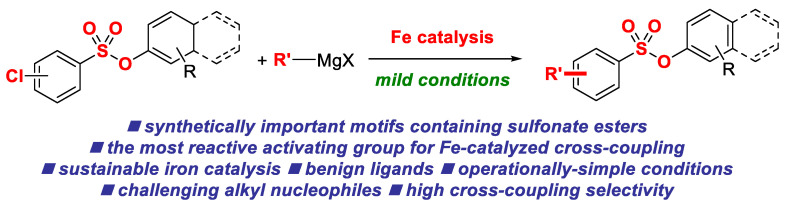
Iron-catalyzed C(sp^2^)–C(sp^3^) cross-coupling of aryl chlorobenzenesulfonates with alkyl Grignard reagents (this study).

Based on recent advances in iron-catalyzed cross-couplings [1,2,3,4,5,6,7,8,9,10,11,12,13] and inspired by our interest in the development of chemoselective methods using iron [21,22,23,24,40], we present our findings on the development of iron-catalyzed C(sp^2^)–C(sp^3^) cross-coupling of aryl chlorobenzenesulfonates with alkyl Grignard reagents (Figure 2). The reaction is characterized by its very good substrate scope, tolerating a range of electronically and sterically varied benzenesulfonates. The protocol operates under very mild, operationally simple conditions using sustainable iron catalysts and is compatible with challenging organometallics possessing β-hydrogens. A notable feature of the protocol is the use of environmentally-friendly cyclic urea ligands in contrast to the reprotoxic NMP [21,41,42]. Perhaps most importantly, the study reveals that aryl sulfonate esters are the most reactive activating groups for iron-catalyzed alkylative C(sp^2^)–C(sp^3^) cross-coupling of aryl chlorides with Grignard reagents [23,43,44,45,46,47,48,49]. This direct iron-catalyzed Kumada cross-coupling, therefore, provides an attractive and highly efficient way for the functionalization of aromatic benzenesulfonates with complete preservation of the aryl sulfonate group. 

## 2. Results

At the outset, we evaluated the model reaction between phenyl 4-chlorobenzenesulfonate and *n*-alkyl Grignard reagent containing β-hydrogens (Table 1). In general, aryl sulfonates contain the SO_2_–O–Ar bond and thus share common features with aryl benzoates bearing the CO–O–Ar bond, which presents a major challenge of cleavage reactions involving the removal of the O–aryl group in both types of esters [50,51]. Thus, in analogy to the cross-coupling of chloro aryl benzoates, where C(acyl)–O bond cleavage was commonly observed in the presence of Grignard reagents [40], similar deconstructive S–O bond cleavage is a common side reaction [50]. On the other hand, it should be considered that using an iron catalyst system with O-coordinating ligands, it is possible to obtain cross-coupling products by activating the C–OSO_2_ bond in aryl tosylates [32,43,52]. Therefore, it was initially unclear if chlorobenzenesulfonatoes can be used effectively as cross-coupling partners in sustainable conditions. The research conducted by Fürstner provides a strong precedent in this regard [43]. Since our focus is on developing operationally practical methods of broad synthetic appeal, we selected rapid addition of Grignard reagent and the use of readily accessible, non-toxic, and environmentally benign O-coordinating ligands. As shown, no reaction was observed in the absence of iron (Table 1, entry 1). Pleasingly, it was found that the reaction with Fe(acac)_3_ afforded the desired cross-coupling product in 73% yield with the remaining mass balance corresponding to hydrolysis and decomposition products (entry 2). Notably, the combined use of iron and cyclic urea (DMI = 1,3-dimethyl-2-imidazolidinone) afforded the alkylated product an excellent yield (Table 1, entries 3–6). Importantly, the use of 20–50 mol% of DMI (entries 3 and 4) resulted in higher yields, while the use of an excess of DMI afforded the cleanest reactions. It should be noted that under these reaction conditions, the cleavage of S–O bond or the formation of unwanted by-products derived from Grignard reagents, including products from β-hydride elimination and homo-coupling, were not observed. Moreover, in contrast to previous studies [23], it is important to point out that the role of the ligand is in facilitating the reaction as decomposition is observed in its absence, while other cross-couplings catalyzed by iron/O-coordinating systems lead to recovered starting materials, indicating an important activating effect of the labile sulfonate ester moiety. 

Having determined that the model aryl chlorobenzenesulfonate serves as an efficient cross-coupling partner under benign iron/DMI conditions, the scope of the optimized iron catalytic system was next explored (Table 2). Pleasingly, we found that electronically-neutral as well as electron-rich aryl 4-chlorobenzenesulfonates, such as 4-*tert*-butyl and 4-methoxy, participated in the chemoselective cross-coupling in excellent yields (98%) (Table 2, entries 1–3). Furthermore, electron-deficient aryl 4-chlorobenzenesulfonates, such as 4-fluoro, are also well-tolerated, and the desired coupling product is obtained in excellent yield (95%, Table 2, entry 4). We also found that both sterically hindered 2-methyl and even 2,6-dimethyl aryl 4-chlorobenzenesulfonates are well-tolerated, affording the products in quantitative yields (Table 2, entries 5–6). Interestingly, using naphthalen-2-yl 4-chlorobenzenesulfonate as a coupling partner under the same reaction conditions, the product was obtained in 50% yield, with the remaining mass balance corresponding to 2-ethylnaphthalene as a result of C–O bond activation. The selectivity of the reaction was improved by reducing the amount of the organomagnesium reagent to 1.05 equiv, which afforded the cross-coupling product in 73% yield (Table 2, entry 7). Fast C–O bond activation in this class of electrophiles is likely due to the electronic conjugation of polyaromatic hydrocarbons compared to phenyl derivatives [52]. Furthermore, an excellent yield (98%) without any modification of the reaction conditions was observed in the cross-coupling of the meta-substituted aryl chloro-benzenesulfonate (entry 8), demonstrating that high coupling efficiency is not dependent on halide conjugation with the sulfonate ester moiety. Pleasingly, we also found that even the sterically hindered ortho-substituted chloro-benzenesulfonate afforded the desired coupling product in 59% yield (entry 9). 

The scope of Grignard reagents was also examined (Table 2, entries 10–13), demonstrating that the cross-coupling of more challenging sterically hindered secondary Grignard reagents is also feasible. Thus, by using alkyl Grignard reagents, such as cyclohexyl (entry 10) and isopropyl (entry 11), the alkylated product was obtained in a 98% yield. The result with *i*-PrMgBr is of particular interest since thus far, this is the highest yield obtained with this Grignard reagent in the iron-catalyzed cross-coupling [21,22,23,24,43,44,45,46,47], which confirms a very high propensity of the sulfonate ester moiety as an activating group in cross-coupling. Furthermore, isomerization of *i*-Pr to *n*-Pr was not observed under the reaction conditions, attesting to the mild nature of this catalytic system (cf. Fe–NHC where isomerization is common) [53]. Finally, we were pleased to find that the challenging phenethyl Grignard reagent, which is prone to β-hydride elimination (entry 12), and sensitive dioxolane Grignard, which serves as a synthetic carbonyl equivalent (entry 13), are also competent nucleophiles in this cross-coupling protocol. It is worthwhile to note that nucleophilic addition to the ester S–O bond was not observed in any of the tested examples, attesting to the mild conditions and facility of the coupling. Furthermore, the observed yields compare very favorably with related iron/NMP-catalyzed Kumada cross-coupling methods using other activating groups, highlighting the potential of aryl sulfonates as versatile activating groups in this cross-coupling manifold.

**Scheme 1:**
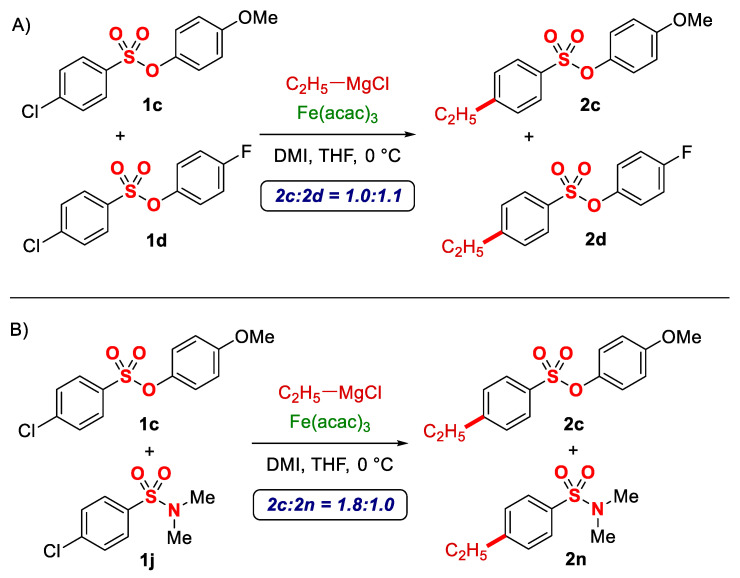
Competition experiments: (**A**) electron-rich vs. electron-deficient benzenesulfonates; (**B**) benzenesulfonates vs. benzenesulfonamides.

Subsequently, intermolecular competition studies were performed to gain insight into the selectivity of this cross-coupling and show the unique reactivity of benzenesulfonates (Scheme 1). Experiments conducted between electron-rich and electron-deficient sulfonates (4-MeO:4-F = 1.0:1.1) revealed that electron-deficient arenes are modestly more reactive (Scheme 1A). Similarly, competition experiments were performed between aryl esters vs. aryl sulfonates, namely, 4-chlorobenzenesulfonate vs. 4-chlorobenzoate (not shown), and in this case, only the alkylated sulfonate was observed. In contrast, the only product observed from aryl benzoate was the nucleophilic addition alcohol product, which shows a marked difference in reactivity of these two classes of compounds. Finally, the excellent activating profile of sulfonates was confirmed in the competition experiments vs. sulfonamides (sulfonate/sulfonamide = 1.8:1) (Scheme 1B); sulfonamides had so far been considered the most activating group for iron-catalyzed cross-couplings [23]. Furthermore, the beneficial use of sulfonates is evident from comparing the reaction yields, which are markedly higher in chlorobenzenesulfonate substrates vs. chlorobenzenesulfobanides [23].

Finally, the effect of various ligand additives on the cross-coupling was investigated (Table 3). One of the greatest limitations in the use of iron catalysts in industrial C(sp^2^)–C(sp^3^) cross-couplings is the need to use reprotoxic NMP [16]. Thus, we investigated the effect of various O-coordinating ligands on the efficiency of the couplings. The conducted study demonstrates that benign cyclic urea ligands such as DMI, DMPU (DMPU = 1,3-dimethyl-3,4,5,6-tetrahydro-2(1*H*)-pyrimidinone), and other cyclic amide ligands such as *N*-methylcaprolactam (N-Me-CPL) show comparable reactivity to NMP (Table 3, entries 1, 2, 4–5). Furthermore, the chelating *N*,*N*-bis(2-methoxyethyl)benzamide that was recently reported by our group [54] appears to be less reactive (Table 3, entry 6); moreover, only with this additive, a small amount of the hydrolysis product was observed. In contrast, piperidinyl benzamide showed comparable reactivity to NMP in this cross-coupling (Table 3, entry 7) [54].

**Scheme 2:**
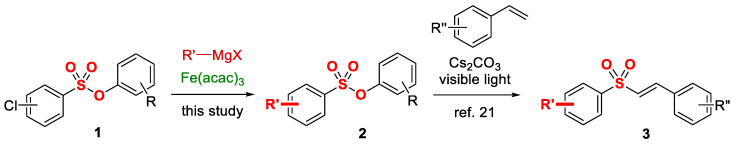
Synthesis of vinyl sulfones.

In a broader synthetic context, despite the limited reactivity of sulfonates with the preservation of organosulfur bonds, such examples have recently started to appear. For example, a recent study described the use of aryl sulfonates as sulfonylation reagents in the preparation of valuable vinyl sulfones [55]. Thus, in combination with an easy and mild way of synthesis various *n*-alkylbenzenesulfonates by iron catalysis, this method represents an important approach in the preparation of *n*-alkylated vinyl arenesulfones, which are valuable reactive intermediates in downstream organic transformations (Scheme 2). 

Several additional points should be noted: (1) Although FeCl_3_ is also effective, the cheap and non-hygroscopic Fe(acac)_3_ is the preferred iron salt from the practical point of view. (2) It is well established that the use of other pseudohalides, such as Br and I, is generally ineffective in this reaction manifold. Triflates can be used; however, these substrates are less preferred due to facile hydrolysis [1,2,3,4,5,6,7,8,9,10,11,12,13,20]. (3) Ortho- and meta-substituted chlorobenzenesulfonates can be used; however, it should be noted that this reactivity is not common, and only a few select activating groups can promote the reactivity of non-conjugated and sterically hindered aryl chlorides in this reaction manifold [1,2,3,4,5,6,7,8,9,10,11,12,13,20,21,22,23,24]. (4) Aryl, benzyl, and alkenyl Grignard reagents are not compatible with this reaction manifold [1,2,3,4,5,6,7,8,9,10,11,12,13,20,21,22,23,24]. (5) NMP is generally required in this reactivity platform, as demonstrated by Cahiez and Fürstner [1,2,3,4,5,6,7,8,9,10,11,12,13,20]. In the present case, it is necessary to use 200 mol% of DMI to obtain the desired product in a high yield. Future studies will likely involve the replacement of NMP with other benign amide-type donors [21,22,23,24].

## 3. Conclusions

In summary, we have reported the iron-catalyzed C(sp^2^)–C(sp^3^) Kumada cross-coupling of chlorobenzenesulfonates with alkyl Grignard reagents. The present study extends the scope of benign iron-catalyzed cross-couplings to chlorobenzenesulfonate electrophiles using benign urea ligands as replacement for toxic NMP. A wide range of electronically and sterically differentiated benzenesulfonates and alkyl organometallics containing β-hydrogens underwent cross-coupling under exceedingly mild reaction conditions with excellent chemoselectivity in the presence of the sensitive sulfonate ester moiety. The developed protocol tolerates an aryl sulfonate ester in the presence of reactive Grignard organometallics without the nucleophilic cleavage of the S–O bond and metal-catalyzed C–O bond activation. Importantly, the study demonstrates that sulfonate esters are so far the most reactive activating groups for iron-catalyzed alkylative C(sp^2^)–C(sp^3^) cross-coupling of aryl chlorides, which is one of the most widely used iron-catalyzed cross-couplings in organic chemistry. Future research should focus on further expanding the scope of iron-catalyzed cross-couplings and the design of new ligands for iron catalysis. 

## 4. Materials and Methods

### 4.1. General Methods

All compounds reported in the manuscript are commercially available or have been previously described in literature unless indicated otherwise. All experiments involving iron were performed using standard Schlenk techniques under argon or nitrogen atmosphere unless stated otherwise. All sulfonate esters have been prepared by standard methods [31]. All yields refer to yields determined by ^1^H NMR and/or GC-MS using an internal standard (optimization) and isolated yields (preparative runs) unless stated otherwise. ^1^H NMR and ^13^C NMR data are given for all compounds in the Experimental Section for characterization purposes. All products have been previously reported unless stated otherwise. All new compounds have been characterized by established guidelines by ^1^H NMR, ^13^C NMR, and HRMS as appropriate. Spectroscopic data matched literature values. General methods have been published [21,22,23,24]. 

### 4.2. General Procedure for Iron-Catalyzed C(sp^2^)–C(sp^3^) Cross-Coupling

An oven-dried vial equipped with a stir bar was charged with a sulfonate ester substrate (neat, typically, 0.50 mmol, 1.0 equiv) and Fe(acac)_3_ (typically, 5 mol%), placed under a positive pressure of argon, and subjected to three evacuation/backfilling cycles under vacuum. Tetrahydrofuran (0.15 M) and ligand were sequentially added with vigorous stirring at room temperature, the reaction mixture was cooled to 0 °C, a solution of Grignard reagent (typically, 1.2 equiv) was added dropwise with vigorous stirring, and the reaction mixture was stirred for the indicated time at 0 °C. After the indicated time, the reaction mixture was diluted with HCl (1.0 *N*, 1.0 mL) and Et_2_O (1 × 30 mL); the organic layer was extracted with HCl (1.0 *N*, 2 × 10 mL), dried, and concentrated. The sample was analyzed by ^1^H NMR (CDCl_3_, 400 MHz) and GC-MS to obtain conversion, yield, and selectivity using internal standard and comparison with authentic samples. Purification by chromatography on silica gel (EtOAc/hexanes = 1/4) afforded the title product.

### 4.3. General Procedure for Determination of Relative Reactivity

According to the general procedure, an oven-dried vial equipped with a stir bar was charged with two chloride substrates (each 0.50 mmol, 1.0 equiv) and Fe(acac)_3_ (5 mol%), placed under a positive pressure of argon and subjected to three evacuation/backfilling cycles under vacuum. Tetrahydrofuran (0.15 M) and DMI (neat, 600 mol%) were sequentially added with vigorous stirring at room temperature, the reaction mixture was cooled to 0 °C, a solution of C_2_H_5_MgCl (2.0 M in THF, 0.25 mmol, 0.50 equiv) was added dropwise with vigorous stirring, and the reaction mixture was stirred for 10 min at 0 °C. Following the standard workup, the sample was analyzed by ^1^H NMR (CDCl_3_, 400 MHz) and GC-MS to obtain conversion, yield, and selectivity using internal standards and comparison with authentic samples.

### 4.4. Characterization Data for Starting Materials

#### 4.4.1. Phenyl 4-chlorobenzenesulfonate (**1a**)

Yield 92% (2.48 g). White solid. ^1^H NMR (400 MHz, CDCl_3_) δ 7.76 (d, *J* = 8.8 Hz, 2H), 7.50 (d, *J* = 8.7 Hz, 2H), 7.34–7.23 (m, 3H), 7.01–6.96 (m, 2H). ^13^C NMR (100 MHz, CDCl_3_) δ 149.59, 141.16, 133.94, 130.09, 129.96, 129.68, 127.56, 122.46. Spectroscopic properties matched those described previously [56].

#### 4.4.2. 4-(tert-Butyl)phenyl 4-chlorobenzenesulfonate (**1b**)

*New compound*. Yield 91% (2.97 g). White solid. Mp = 95–96 °C. ^1^H NMR (400 MHz, CDCl_3_) δ 7.78 (d, *J* = 8.7 Hz, 2H), 7.50 (d, *J* = 8.7 Hz, 2H), 7.30 (d, *J* = 8.8 Hz, 2H), 6.89 (d, *J* = 8.8 Hz, 2H), 1.28 (s, 9H). ^13^C NMR (100 MHz, CDCl_3_) δ 150.59, 147.23, 141.00, 134.19, 130.06, 129.63, 126.82, 121.73, 34.73, 31.45. HRMS (ESI/Q-TOF) *m*/*z*: [M + Na]^+^ calcd for C_16_H_17_ClO_3_SNa 347.0485 found 347.0469.

#### 4.4.3. 4-Methoxyphenyl 4-chlorobenzenesulfonate (**1c**)

Yield 95% (2.84 g). White solid. ^1^H NMR (400 MHz, CDCl_3_) δ 7.74 (d, *J* = 8.8 Hz, 2H), 7.49 (d, *J* = 8.8 Hz, 2H), 6.88 (d, *J* = 9.2 Hz, 2H), 6.78 (d, *J* = 9.2 Hz, 2H), 3.77 (s, 3H). ^13^C NMR (100 MHz, CDCl_3_) δ 158.51, 142.95, 141.07, 133.84, 130.13, 129.62, 123.42, 114.75, 55.73. Spectroscopic properties matched those described previously [29].

#### 4.4.4. 4-Fluorophenyl 4-chlorobenzenesulfonate (**1d**)

*New compound.* Yield 92% (2.63 g). White solid. Mp = 87–88 °C. ^1^H NMR (400 MHz, CDCl_3_) δ 7.76 (d, *J* = 8.7 Hz, 2H), 7.52 (d, *J* = 8.7 Hz, 2H), 7.03–6.93 (m, 4H). ^13^C NMR (100 MHz, CDCl_3_) δ 162.52, 160.06, 145.32, 145.30, 141.38, 133.56, 130.10, 129.77, 124.11 (d, *J*^F^ = 8.8 Hz), 116.71 (d, *J*^F^ = 23.7 Hz). HRMS (ESI/Q-TOF) *m*/*z*: [M + Na]^+^ calcd for C_12_H_8_ClFO_3_SNa 308.9764 found 308.9753.

#### 4.4.5. o-Tolyl 4-chlorobenzenesulfonate (**1e**)

*New compound.* Yield 95% (2.69 g). White solid. Mp = 54–55 °C. ^1^H NMR (400 MHz, CDCl_3_) δ 7.81 (d, *J* = 8.8 Hz, 2H), 7.52 (d, *J* = 8.8 Hz, 2H), 7.20–7.11 (m, 3H), 7.00–6.95 (m, 1H), 2.09 (s, 3H). ^13^C NMR (100 MHz, CDCl_3_) δ 148.32, 141.15, 134.74, 131.93, 131.66, 129.98, 129.73, 127.43, 127.23, 122.32, 16.50. HRMS (ESI/Q-TOF) *m*/*z*: [M + Na]^+^ calcd for C_13_H_11_ClO_3_SNa 305.0015 found 305.0010.

#### 4.4.6. 2,6-Dimethylphenyl 4-chlorobenzenesulfonate (**1f**)

*New compound.* Yield 93% (2.76 g). White solid. Mp = 53–54 °C. ^1^H NMR (400 MHz, CDCl_3_) δ 7.91 (d, *J* = 8.7 Hz, 2H), 7.55 (d, *J* = 8.7 Hz, 2H), 7.10–7.00 (m, 3H), 2.13 (s, 6H). ^13^C NMR (100 MHz, CDCl_3_) δ 147.56, 140.93, 135.93, 132.20, 129.76, 129.63, 129.49, 127.06, 17.50. HRMS (ESI/Q-TOF) *m*/*z*: [M + Na]^+^ calcd for C_14_H_13_ClO_3_SNa 319.0172 found 319.0171.

#### 4.4.7. Naphthalen-2-yl 4-chlorobenzenesulfonate (**1g**)

*New compound.* Yield 95% (3.04 g). White solid. Mp = 129–130 °C. ^1^H NMR (400 MHz, CDCl_3_) δ 7.84–7.73 (m, 5H), 7.53–7.45 (m, 5H), 7.09 (dd, *J* = 8.9, 2.4 Hz, 1H). ^13^C NMR (100 MHz, CDCl_3_) δ 147.09, 141.20, 133.95, 133.54, 132.11, 130.15, 130.12, 129.71, 128.06, 127.95, 127.20, 126.76, 121.07, 120.09. HRMS (ESI/Q-TOF) *m*/*z*: [M + Na]^+^ calcd for C_16_H_11_ClO_3_SNa 341.0015 found 341.0025.

#### 4.4.8. 4-Methoxyphenyl 3-chlorobenzenesulfonate (**1h**)

*New compound.* Yield 96% (2.88 g). Light beige solid. Mp = 68–69 °C. ^1^H NMR (400 MHz, CDCl_3_) δ 7.84–7.81 (m, 1H), 7.71–7.66 (m, 1H), 7.66–7.61 (m, 1H), 7.47 (t, *J* = 8.0 Hz, 1H), 6.90 (d, *J* = 9.2 Hz, 2H), 6.79 (d, *J* = 9.2 Hz, 2H), 3.78 (s, 3H). ^13^C NMR (100 MHz, CDCl_3_) δ 158.56, 142.88, 137.06, 135.54, 134.48, 130.53, 128.63, 126.82, 123.37, 114.78, 55.74. HRMS (ESI/Q-TOF) *m*/*z*: [M + Na]^+^ calcd for C_13_H_11_ClO_4_SNa 320.9964 found 320.9975.

#### 4.4.9. 4-Methoxyphenyl 2-chlorobenzenesulfonate (**1i**)

*New compound.* Yield 97% (2.90 g). Light beige solid. Mp = 64–65 °C. ^1^H NMR (400 MHz, CDCl_3_) δ 7.91–7.86 (m, 1H), 7.64–7.53 (m, 2H), 7.38–7.31 (m, 1H), 7.05–6.98 (m, 2H), 6.79–6.74 (m, 2H), 3.74 (s, 3H). ^13^C NMR (100 MHz, CDCl_3_) δ 158.47, 142.87, 135.31, 133.44, 133.34, 132.68, 132.25, 127.22, 123.13, 114.75, 55.68. HRMS (ESI/Q-TOF) *m*/*z*: [M + Na]^+^ calcd for C_13_H_11_ClO_4_SNa 320.9964 found 320.9970.

### 4.5. Characterization Data for Cross-Coupling Products

#### 4.5.1. Phenyl 4-ethylbenzenesulfonate (**2a**)

*New compound.* Prepared according to the general procedure using phenyl 4-chlorobenzenesulfonate (0.50 mmol), Fe(acac)_3_ (5 mol%), DMI (600 mol%), THF (0.15 M), and C_2_H_5_MgCl (2.0 M in THF, 1.20 equiv). The reaction mixture was stirred for 10 min at 0 °C. Yield 98% (128.4 mg). Colorless oil. ^1^H NMR (400 MHz, CDCl_3_) δ 7.73 (d, *J* = 8.4 Hz, 2H),7.36–7.21 (m, 5H), 7.01–6.96 (m, 2H), 2.74 (q, *J* = 7.6 Hz, 2H), 1.27 (t, *J* = 7.6 Hz, 3H). ^13^C NMR (100 MHz, CDCl_3_) δ 151.57, 149.79, 132.69, 129.76, 128.78, 128.74, 127.24, 122.57, 29.08, 15.17. HRMS (ESI/Q-TOF) *m*/*z*: [M + Na]^+^ calcd for C_14_H_14_O_3_SNa 285.0561 found 285.0557.

#### 4.5.2. 4-(tert-Butyl)phenyl 4-ethylbenzenesulfonate (**2b**)

*New compound.* Prepared according to the general procedure using 4-(*tert*-butyl)phenyl 4-chlorobenzenesulfonate (0.50 mmol), Fe(acac)_3_ (5 mol%), DMI (600 mol%), THF (0.15 M), and C_2_H_5_MgCl (2.0 M in THF, 1.20 equiv). The reaction mixture was stirred for 10 min at 0 °C. Yield 98% (156.2 mg). Colorless oil. ^1^H NMR (400 MHz, CDCl_3_) δ 7.75 (d, *J* = 8.4 Hz, 2H), 7.34 (d, *J* = 8.5 Hz, 2H), 7.28 (d, *J* = 8.9 Hz, 2H), 6.90 (d, *J* = 8.9 Hz, 2H), 2.74 (q, *J* = 7.6 Hz, 2H), 1.30–1.25 (m, 12H). ^13^C NMR (100 MHz, CDCl_3_) δ 151.42, 150.22, 147.44, 132.99, 128.72, 128.70, 126.64, 121.83, 34.69, 31.46, 29.07, 15.18. HRMS (ESI/Q-TOF) *m*/*z*: [M + Na]^+^ calcd for C_18_H_22_O_3_SNa 341.1187 found 341.1185.

#### 4.5.3. 4-Methoxyphenyl 4-ethylbenzenesulfonate (**2c**)

*New compound.* Prepared according to the general procedure using 4-methoxyphenyl 4-chlorobenzenesulfonate (0.50 mmol), Fe(acac)_3_ (5 mol%), DMI (600 mol%), THF (0.15 M), and C_2_H_5_MgCl (2.0 M in THF, 1.20 equiv). The reaction mixture was stirred for 10 min at 0 °C. Yield 98% (143.1 mg). Colorless oil. ^1^H NMR (400 MHz, CDCl_3_) δ 7.71 (d, *J* = 8.4 Hz, 2H), 7.33 (d, *J* = 8.5 Hz, 2H), 6.88 (d, *J* = 9.2 Hz, 2H), 6.76 (d, *J* = 9.2 Hz, 2H), 3.76 (s, 3H), 2.74 (q, *J* = 7.6 Hz, 2H), 1.26 (t, *J* = 7.6 Hz, 3H). ^13^C NMR (100 MHz, CDCl_3_) δ 158.30, 151.48, 143.17, 132.56, 128.80, 128.69, 123.50, 114.57, 55.68, 29.05, 15.14. HRMS (ESI/Q-TOF) *m*/*z*: [M + Na]^+^ calcd for C_15_H_16_O_4_SNa 315.0667 found 315.0657.

#### 4.5.4. 4-Fluorophenyl 4-ethylbenzenesulfonate (**2d**)

*New compound.* Prepared according to the general procedure using 4-fluorophenyl 4-chlorobenzenesulfonate (0.50 mmol), Fe(acac)_3_ (5 mol%), DMI (600 mol%), THF (0.15 M), and C_2_H_5_MgCl (2.0 M in THF, 1.20 equiv). The reaction mixture was stirred for 10 min at 0 °C. Yield 95% (133.2 mg). Colorless oil. ^1^H NMR (400 MHz, CDCl_3_) δ 7.72 (d, *J* = 8.5 Hz, 2H), 7.34 (d, *J* = 8.6 Hz, 2H), 7.00–6.92 (m, 4H), 2.75 (q, *J* = 7.6 Hz, 2H), 1.27 (t, *J* = 7.6 Hz, 3H). ^13^C NMR (100 MHz, CDCl_3_) δ 162.38, 159.93, 151.81, 145.56, 145.53, 132.28, 128.82, 128.81, 124.20 (d, *J*^F^ = 8.8 Hz), 116.49 (d, *J*^F^ = 23.7 Hz). HRMS (ESI/Q-TOF) *m*/*z*: [M + Na]^+^ calcd for C_14_H_13_FO_3_SNa 303.0467 found 303.0466.

#### 4.5.5. o-Tolyl 4-ethylbenzenesulfonate (**2e**)

*New compound.* Prepared according to the general procedure using *o*-tolyl 4-chlorobenzenesulfonate (0.50 mmol), Fe(acac)_3_ (5 mol%), DMI (600 mol%), THF (0.15 M), and C_2_H_5_MgCl (2.0 M in THF, 1.20 equiv). The reaction mixture was stirred for 10 min at 0 °C. Yield 98% (135.2 mg). Colorless oil. ^1^H NMR (400 MHz, CDCl_3_) δ 7.77 (d, *J* = 8.5 Hz, 2H), 7.34 (d, *J* = 8.6 Hz, 2H), 7.18–7.08 (m, 3H), 7.02–6.97 (m, 1H), 2.75 (q, *J* = 7.6 Hz, 2H), 2.07 (s, 3H), 1.27 (t, *J* = 7.6 Hz, 3H). ^13^C NMR (100 MHz, CDCl_3_) δ 151.58, 148.47, 133.45, 131.77, 131.71, 128.79, 128.64, 127.12, 127.05, 122.49, 29.08, 16.42, 15.23. HRMS (ESI/Q-TOF) *m*/*z*: [M + Na]^+^ calcd for C_15_H_16_O_3_SNa 299.0718 found 299.0712.

#### 4.5.6. 2,6-Dimethylphenyl 4-ethylbenzenesulfonate (**2f**)

*New compound.* Prepared according to the general procedure using 2,6-dimethylphenyl 4-chlorobenzenesulfonate (0.50 mmol), Fe(acac)_3_ (5 mol%), DMI (600 mol%), THF (0.15 M), and C_2_H_5_MgCl (2.0 M in THF, 1.20 equiv). The reaction mixture was stirred for 10 min at 0 °C. Yield 97% (140.5 mg). Colorless oil. ^1^H NMR (400 MHz, CDCl_3_) δ 7.88 (d, *J* = 8.4 Hz, 2H), 7.38 (d, *J* = 8.5 Hz, 2H), 7.10–6.99 (m, 3H), 2.76 (q, *J* = 7.6 Hz, 2H), 2.13 (s, 6H), 1.28 (t, *J* = 7.6 Hz, 3H). ^13^C NMR (100 MHz, CDCl_3_) δ 151.45, 147.64, 134.64, 132.40, 129.36, 128.85, 128.35, 126.81, 29.10, 17.45, 15.30. HRMS (ESI/Q-TOF) *m*/*z*: [M + Na]^+^ calcd for C_16_H_18_O_3_SNa 313.0874 found 313.0876.

#### 4.5.7. Naphthalen-2-yl 4-ethylbenzenesulfonate (**2g**)

*New compound.* Prepared according to the general procedure using naphthalen-2-yl 4-chlorobenzenesulfonate (0.50 mmol), Fe(acac)_3_ (5 mol%), DMI (600 mol%), THF (0.15 M), and C_2_H_5_MgCl (2.0 M in THF, 1.05 equiv). The reaction mixture was stirred for 10 min at 0 °C. Yield 73% (114.2 mg). White solid. Mp = 75–76 °C. ^1^H NMR (400 MHz, CDCl_3_) δ 7.84–7.78 (m, 1H), 7.77–7.70 (m, 4H), 7.52–7.44 (m, 3H), 7.32 (d, *J* = 8.6 Hz, 2H), 7.10 (dd, *J* = 8.9, 2.4 Hz, 1H), 2.73 (q, *J* = 7.6 Hz, 2H), 1.26 (t, *J* = 7.6 Hz, 3H). ^13^C NMR (100 MHz, CDCl_3_) δ 151.64, 147.34, 133.58, 132.69, 132.03, 129.90, 128.84, 128.79, 128.03, 127.91, 127.01, 126.54, 121.37, 120.14, 29.10, 15.20. HRMS (ESI/Q-TOF) *m*/*z*: [M + Na]^+^ calcd for C_18_H_16_O_3_SNa 335.0718 found 335.0711.

#### 4.5.8. 4-Methoxyphenyl 3-ethylbenzenesulfonate (**2h**)

*New compound.* Prepared according to the general procedure using 4-methoxyphenyl 3-chlorobenzenesulfonate (0.50 mmol), Fe(acac)_3_ (5 mol%), DMI (600 mol%), THF (0.15 M), and C_2_H_5_MgCl (2.0 M in THF, 1.20 equiv). The reaction mixture was stirred for 10 min at 0 °C. Yield 98% (142.8 mg). Light beige oil. ^1^H NMR (400 MHz, CDCl_3_) δ 7.65–7.60 (m, 2H), 7.50–7.46 (m, 1H), 7.44–7.39 (m, 1H), 6.87 (d, *J* = 9.3 Hz, 2H), 6.77 (d, *J* = 9.2 Hz, 2H), 3.76 (s, 3H), 2.70 (q, *J* = 7.6 Hz, 2H), 1.22 (t, *J* = 7.6 Hz, 3H). ^13^C NMR (100 MHz, CDCl_3_) δ 158.36, 145.83, 143.18, 135.26, 134.01, 129.19, 127.91, 126.04, 123.52, 114.59, 55.71, 28.75, 15.46. HRMS (ESI/Q-TOF) *m*/*z*: [M + Na]^+^ calcd for C_15_H_16_O_4_SNa 315.0667 found 315.0659.

#### 4.5.9. 4-Methoxyphenyl 2-ethylbenzenesulfonate (**2i**)

*New compound.* Prepared according to the general procedure using 4-methoxyphenyl 2-chlorobenzenesulfonate (0.50 mmol), Fe(acac)_3_ (5 mol%), DMI (600 mol%), THF (0.15 M), and C_2_H_5_MgCl (2.0 M in THF, 1.20 equiv). The reaction mixture was stirred for 60 min at 0 °C. Yield 59% (85.7 mg). Colorless oil. ^1^H NMR (400 MHz, CDCl_3_) δ 7.78 (dd, *J* = 8.0, 1.3 Hz, 1H), 7.58 (td, *J* = 7.6, 1.4 Hz, 1H), 7.48–7.45 (m, 1H), 7.29–7.23 (m, 1H), 6.87 (d, *J* = 9.2, 2H), 6.75 (d, *J* = 9.2, 2H), 3.75 (s, 3H), 3.18 (q, *J* = 7.5 Hz, 2H), 1.36 (t, *J* = 7.5 Hz, 3H). ^13^C NMR (100 MHz, CDCl_3_) δ 158.32, 144.97, 143.08, 134.43, 133.79, 131.06, 130.87, 126.21, 123.31, 114.66, 55.70, 26.36, 15.43. HRMS (ESI/Q-TOF) *m*/*z*: [M + Na]^+^ calcd for C_15_H_16_O_4_SNa 315.0667 found 315.0663.

#### 4.5.10. 4-Methoxyphenyl 4-cyclohexylbenzenesulfonate (**2j**)

*New compound.* Prepared according to the general procedure using 4-methoxyphenyl 4-chlorobenzenesulfonate (0.50 mmol), Fe(acac)_3_ (5 mol%), DMI (600 mol%), THF (0.15 M), and *c-*C_6_H_11_MgCl (1.0 M in THF, 1.20 equiv). The reaction mixture was stirred for 60 min at 0 °C. Yield 98% (169.4 mg). Colorless oil. ^1^H NMR (400 MHz, CDCl_3_) δ 7.72 (d, *J* = 8.5 Hz, 2H), 7.33 (d, *J* = 8.3 Hz, 2H), 6.88 (d, *J* = 9.2 Hz, 2H), 6.77 (d, *J* = 9.2 Hz, 2H), 3.77 (s, 3H), 2.65–2.53 (m, 1H), 1.93–1.82 (m, 4H), 1.81–1.72 (m, 1H), 1.49–1.33 (m, 4H), 1.31–1.22 (m, 1H). ^13^C NMR (100 MHz, CDCl_3_) δ 158.31, 155.19, 143.20, 132.64, 128.80, 127.71, 123.55, 114.58, 55.72, 44.78, 34.19, 26.76, 26.06. HRMS (ESI/Q-TOF) *m*/*z*: [M + Na]^+^ calcd for C_19_H_22_O_4_SNa 369.1137 found 369.1131.

#### 4.5.11. 4-Methoxyphenyl 4-isopropylbenzenesulfonate (**2k**)

*New compound.* Prepared according to the general procedure using 4-methoxyphenyl 4-chlorobenzenesulfonate (0.50 mmol), Fe(acac)_3_ (5 mol%), DMI (600 mol%), THF (0.15 M), and *i*-PrMgBr (1.0 M in THF, 1.20 equiv). The reaction mixture was stirred for 60 min at 0 °C. Yield 98% (150.0 mg). Colorless oil. ^1^H NMR (400 MHz, CDCl_3_) δ 7.73 (d, *J* = 8.5 Hz, 2H), 7.36 (d, *J* = 8.3 Hz, 2H), 6.89 (d, *J* = 9.2 Hz, 2H), 6.77 (d, *J* = 9.2 Hz, 2H), 3.76 (s, 3H), 3.05–2.92 (m, 1H), 1.27 (d, *J* = 6.9 Hz, 6H). ^13^C NMR (100 MHz, CDCl_3_) δ 158.31, 156.06, 143.19, 132.71, 128.84, 127.34, 123.53, 114.58, 55.71, 34.42, 23.74. HRMS (ESI/Q-TOF) *m*/*z*: [M + Na]^+^ calcd for C_16_H_18_O_4_SNa 329.0824 found 329.0811.

#### 4.5.12. 4-Methoxyphenyl 4-phenethylbenzenesulfonate (**2l**)

*New compound.* Prepared according to the general procedure using 4-methoxyphenyl 4-chlorobenzenesulfonate (0.50 mmol), Fe(acac)_3_ (5 mol%), DMI (600 mol%), THF (0.15 M), and PhCH_2_CH_2_MgCl (1.0 M in THF, 2.00 equiv). The reaction mixture was stirred for 60 min at 0 °C. Yield 98% (180.2 mg). White solid. Mp = 93–94 °C. ^1^H NMR (400 MHz, CDCl_3_) δ 7.67 (d, *J* = 8.4 Hz, 2H), 7.31–7.17 (m, 5H), 7.13–7.07 (m, 2H), 6.85 (d, *J* = 9.3 Hz, 2H), 6.76 (d, *J* = 9.2 Hz, 2H), 3.76 (s, 3H), 3.03–2.97 (m, 2H), 2.96–2.91 (m, 2H). ^13^C NMR (100 MHz, CDCl_3_) δ 158.32, 148.84, 143.15, 140.61, 132.80, 129.40, 128.76, 128.62, 128.58, 126.42, 123.52, 114.57, 55.70, 37.88, 37.33. HRMS (ESI/Q-TOF) *m*/*z*: [M + Na]^+^ calcd for C_21_H_20_O_4_SNa 391.0980 found 391.0968.

#### 4.5.13. 4-Methoxyphenyl 4-(2-(1,3-dioxan-2-yl)ethyl)benzenesulfonate (**2m**)

*New compound.* Prepared according to the general procedure using 4-methoxyphenyl 4-chlorobenzenesulfonate (0.50 mmol), Fe(acac)_3_ (5 mol%), DMI (600 mol%), THF (0.15 M), and (2-(1,3-dioxan-2-yl)ethyl)magnesium bromide (0.5 M in THF, 3.00 equiv). The reaction mixture was stirred for 15 h at 23 °C. Yield 98% (184.7 mg). Colorless oil. ^1^H NMR (400 MHz, CDCl_3_) δ 7.71 (d, *J* = 8.4 Hz, 2H), 7.33 (d, *J* = 8.5 Hz, 2H), 6.87 (d, *J* = 9.2 Hz, 2H), 6.76 (d, *J* = 9.2 Hz, 2H), 4.50 (t, *J* = 5.1 Hz, 1H), 4.16–4.08 (m, 2H), 3.80–3.69 (m, 5H), 2.85–2.76 (m, 2H), 2.15–2.02 (m, 1H), 1.96–1.88 (m, 2H), 1.40–1.33 (m, 1H). ^13^C NMR (100 MHz, CDCl_3_) δ 158.31, 149.22, 143.13, 132.79, 129.26, 128.83, 123.49, 114.57, 100.99, 67.04, 55.68, 36.13, 30.14, 25.87. HRMS calcd for C_19_H_22_O_6_SNa (M^+^ + Na) 401.1035 found 401.1039. 

## Figures and Tables

**Table 1:**
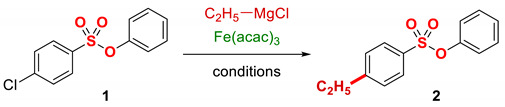
Optimization of Fe-catalyzed cross-coupling ^1^.

Entry	Fe(acac)_3_ (mol%)	Ligand	mol%	Time	Yield (%) ^2^
1	-	-	-	10 min	0
2	5	-	-	10 min	73
3	5	DMI	20	10 min	81
4	5	DMI	50	10 min	87
5	5	DMI	200	10 min	98
6 ^3^	5	DMI	600	10 min	>98

^1^ Conditions: **1** (0.50 mmol), Fe(acac)_3_ (5 mol%), THF (0.15 M), C_2_H_5_MgCl (1.20 equiv, 2.0 M, THF), 0 °C, 10 min. RMgCl added dropwise over 1–2 s. ^2^ Determined by ^1^H NMR and/or GC-MS. ^3^ Note: >98% yield using EtMgBr instead of EtMgCl; >98% yield using FeCl_3_ instead of Fe(acac)_3_.

**Table 2:**
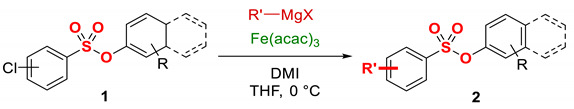
Iron-catalyzed C(sp^2^)–C(sp^3^) cross-coupling of aryl chlorobenzenesulfonates with alkyl Grignard reagents ^1^.

Entry	Substrate	2	Product	Yield (%)
1	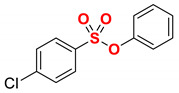	**2a**	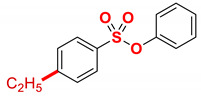	98
2	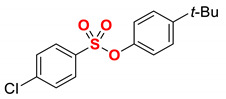	**2b**	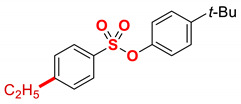	98
3	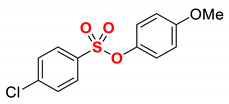	**2c**	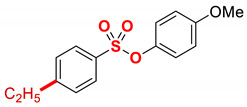	98
4	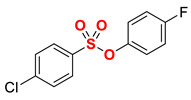	**2d**	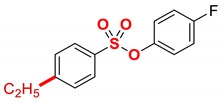	95
5	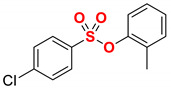	**2e**	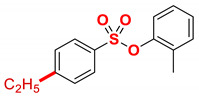	98
6	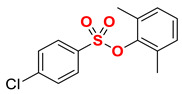	**2f**	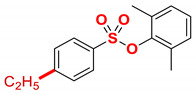	97
7 ^2^	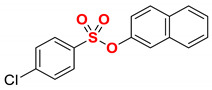	**2g**	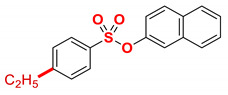	73
8	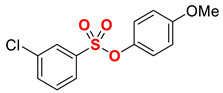	**2h**	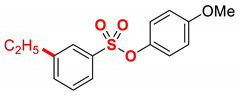	98
9 ^3^	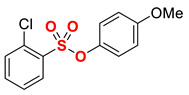	**2i**	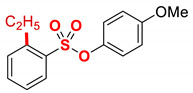	59
10 ^3^	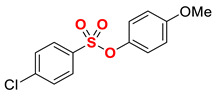	**2j**	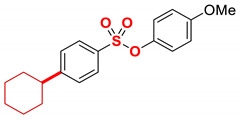	98
11 ^3^	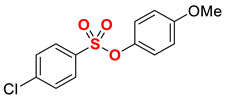	**2k**	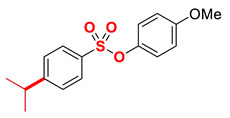	98
12 ^4^	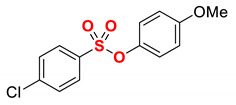	**2l**	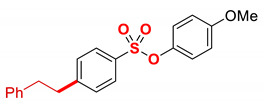	98
13 ^5^	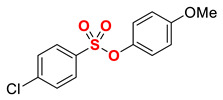	**2m**	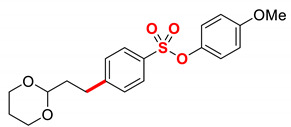	98

^1^ Conditions: **1** (0.50 mmol), Fe(acac)_3_ (5 mol%), THF (0.15 M), DMI (600 mol%), RMgX (1.20 equiv, THF), 0 °C, 10 min. ^2^ RMgX (1.05 equiv). ^3^ 1 h, ^4^ 1 h, RMgX (2.0 equiv), ^5^ 15 h, 23 °C, RMgX (3.0 equiv). See the Supplementary Materials for details.

**Table 3:**
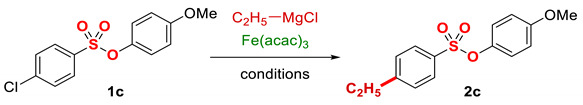
O-Coordinating ligand effect on iron-catalyzed cross-coupling of aryl chlorobenzenesulfonates ^1^.

Entry	Fe(acac)_3_ (mol%)	Ligand	mol%	Time (min)	Yield (%) ^2^
1	5	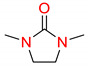	600	10	>98
2	5	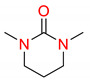	600	10	>98
3	5	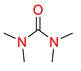	600	10	94
4	5	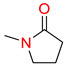	600	10	>98
5	5	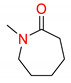	600	10	97
6	5	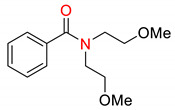	600	10	78
7	5	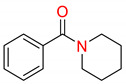	600	10	94

^1^ Conditions: **1** (0.50 mmol), Fe(acac)_3_ (5 mol%), THF (0.15 M), C_2_H_5_MgCl (1.20 equiv, 2.0 M, THF), 0 °C, 10 min. RMgCl added dropwise over 1–2 s. ^2^ Determined by ^1^H NMR and/or GC-MS.

## Data Availability

Not applicable.

## Supplementary Materials

^1^H and ^13^C NMR spectra are available online.

## Sample Availability

Not available.

## References

[1] Molander G.A., Wolfe J.P., Larhed M. (2013). Science of Synthesis: Cross-Coupling and Heck-Type Reactions.

[2] de Meijere A., Bräse S., Oestreich M. (2014). Metal-Catalyzed Cross-Coupling Reactions and More.

[3] Colacot T.J. (2015). New Trends in Cross-Coupling.

[4] Fürstner A., Martin R. (2005). Advances in Iron Catalyzed Cross Coupling Reactions. Chem. Lett..

[5] Sherry B.D., Fürstner A. (2008). The Promise and Challenge of Iron-Catalyzed Cross Coupling. Acc. Chem. Res..

[6] Czaplik W.M., Mayer M., Cvengros J., Jacobi von Wangelin A. (2009). Coming of Age: Sustainable Iron-Catalyzed Cross-Coupling Reactions. ChemSusChem.

[7] Plietker B. (2011). Topic in Organometallic Chemistry Also Available Electronically. Iron Catalysis–Fundamentals and Applications.

[8] Bauer E.B. (2015). Iron Catalysis II. Top. Organomet. Chem..

[9] Marek I., Rappoport Z. (2014). The Chemistry of Organoiron Compounds.

[10] Bauer I., Knölker H.J. (2015). Iron Catalysis in Organic Synthesis. Chem. Rev..

[11] Bakas N.J., Neidig M.L. (2021). Additive and Counterion Effects in Iron-Catalyzed Reactions Relevant to C-C Bond Formation. ACS Catal..

[12] Fürstner A. (2016). Base-Metal Catalysis Marries Utilitarian Aspects with Academic Fascination. Adv. Synth. Catal..

[13] Ludwig J.R., Schindler C.S. (2017). Catalyst: Sustainable Catalysis. Chem.

[14] Giri R., Thapa S., Kafle A. (2014). Palladium- Catalysed, Directed C-H Coupling with Organometallics. Adv. Synth. Catal..

[15] Jana R., Pathak T.P., Sigman M.S. (2011). Advances in Transition Metal (Pd,Ni,Fe)-Catalyzed Cross-Coupling Reactions Using Alkyl-organometallics as Reaction Partners. Chem. Rev..

[16] Piontek A., Bisz E., Szostak M. (2018). Iron-Catalyzed Cross-Coupling in the Synthesis of Pharmaceuticals: In Pursuit of Sustainability. Angew. Chem. Int. Ed..

[17] Roughley S.D., Jordan A.M. (2011). The Medicinal Chemist’s Toolbox: An Analysis of Reactions Used in the Pursuit of Drug Candidates. J. Med. Chem..

[18] Brown D.G., Bostrçm J. (2016). Analysis of Past and Present Synthetic Methodologies in Medicinal Chemistry: Where Have All the New Reactions Gone?. J. Med. Chem..

[19] Schneider N., Lowe D.M., Sayle R.A., Tarselli M.A., Landrum G.A. (2016). Big Data from Pharmaceuticals Patents: A Computational Analysis of Medicinal Chemists’ Bread and Butter. J. Med. Chem..

[20] Cahiez G., Avedissian H. (1998). Highly Stereo- and Chemoselective Iron-Catalyzed Alkenylation of Organomagnesium Compounds. Synthesis.

[21] Bisz E., Szostak M. (2017). Cyclic Ureas (DMI, DMPU) as Efficient, Sustainable Ligands in Iron-Catalyzed C(sp^2^)–C(sp^3^) Coupling of Aryl Chlorides and Tosylates. Green Chem..

[22] Bisz E., Szostak M. (2018). 2-Methyltetrahydrofuran: A Green Solvent for Iron-Catalyzed Cross-Coupling Reactions. ChemSusChem.

[23] Bisz E., Szostak M. (2018). Iron-Catalyzed C(sp^2^)–C(sp^3^) Cross-Coupling of Chlorobenzenesulfonamides with Alkyl Grignard Reagents: Entry to Alkylated Aromatics. J. Org. Chem..

[24] Bisz E., Szostak M. (2019). Iron-Catalyzed C(sp^2^)–C(sp^3^) Cross-Coupling of Chlorobenzamides with Alkyl Grignard Reagents: Development of Catalyst System, Synthetic Scope and Application. Adv. Synth. Catal..

[25] Yan L., Müller C.E. (2004). Preparation, Properties, Reactions, and Adenosine Receptor Affinities of Sulfophenylxanthine Nitrophenyl Esters:  Toward the Development of Sulfonic Acid Prodrugs with Peroral Bioavailability. J. Med. Chem..

[26] Zuse A., Schmidt P., Baasner S., Böhm K.J., Müller K., Gerlach M., Günther E.G., Unger E., Prinz H. (2007). Sulfonate Derivatives of Naphtho[2,3-b]thiophen-4(9H)-one and 9(10H)-anthracenone as Highly Active Antimicrotubule Agents. Synthesis, Antiproliferative Activity, and Inhibition of Tubulin Polymerization. J. Med. Chem..

[27] Park J.-H., Lee G.-E., Lee S.-D., Hien T.T., Kim S., Yang J.W., Cho J.-H., Ko H., Lim S.-C., Kim Y.-G. (2015). Discovery of Novel 2,5-dioxoimidazolidine-based P2X(7) Receptor Antagonists as Constrained Analogues of KN62. J. Med. Chem..

[28] Pisani L., Barletta M., Soto- Otero R., Nicolotti O., Mendez-Alvarez E., Catto M., Introcaso A., Stefanachi A., Cellamare S., Altomare C. (2013). Discovery, Biological Evaluation, and Structure-Activity and -Selectivity Relationships of 6′-Substituted (E)-2-(Benzofuran3(2H)-ylidene)-N-methylacetamides, a Novel Class of Potent and Selective Monoamine Oxidase Inhibitors. J. Med. Chem..

[29] Betts L.M., Tam N.C., Kabir S.M., Langer R.F., Crandall I. (2006). Ether Aryl Sylfonic acid Esters with Improved Anticancer/Antimalarial Activities. Aust. J. Chem..

[30] Wuts P.G.M., Greene T.W. (2007). Greene’s Protective Groups in Organic Synthesis.

[31] Alam M.S., Koo S. (2018). Deprotection of Durable Benzenesulfonyl Protection for Phenols-Efficient Synthesis of Polyphenols. Synth. Commun..

[32] Bisz E., Szostak M. (2017). Iron-Catalyzed C-O Bond Activation: Opportunity for Sustainable Catalysis. ChemSusChem.

[33] Zhou T., Szostak M. (2020). Palladium-Catalyzed Cross-Couplings by C–O Bond Activation. Catal. Sci. Technol..

[34] Wang Q., Dai Z., Di X., Huang Q., Wang Y., Zhu J. (2020). Pd(NHC)(cinnamyl)Cl-Catalyzed Suzuki Cross-Coupling Reaction of Aryl Sulfonates with Arylboronic Acids. Mol. Divers..

[35] Piontek A., Ochędzan-Siodłak W., Bisz E., Szostak M. (2019). Nickel-Catalyzed C(*sp*^2^)−C(*sp*^3^) Kumada Cross-Coupling of Aryl Tosylates with Alkyl Grignard Reagents. Adv. Synth. Catal..

[36] Piontek A., Ochędzan-Siodłak W., Bisz E., Szostak M. (2021). Cobalt-NHC Catalyzed C(sp^2^)−C(sp^3^) and C(sp^2^)−C(sp^2^) Kumada Cross-Coupling of Aryl Tosylates with Alkyl and Aryl Grignard Reagents. ChemCatChem.

[37] Ahmed N., Dubuc C., Rousseau J., Benard F., van Lier J. (2007). Synthesis, Characterization, and Estrogen Receptor Binding Affinity of Flavone-, Indole-, and Furan-estradiol Conjugates. Bioorg. Med. Chem. Lett..

[38] Bensulong S., Boonsombat J., Ruchirawat S. (2013). DBU-mediated Regioselective Intramolecular Cyclization/Dehydration of Ortho Diketo Phenoxyethers: A Synthesis of 2,3-substituted g-benzopyranones. Tetrahedron.

[39] Avitabile B.G., Smith C.A., Judd D.B. (2005). Pentafluorophenyl Sulfonate Ester as a Protecting Group for the Preparation of Biaryl- and Heterobiaryl Sulfonate Esters. Org. Lett..

[40] Bisz E., Szostak M. (2020). Iron- Catalyzed C(sp^2^)–C(sp^3^) Cross-Coupling of Aryl Chlorobenzoates with Alkyl Grignard Reagents. Molecules.

[41] Jesso P.G. (2011). Searching for green solvents. Green Chem..

[42] Welton T. (2015). Solvents and sustainable chemistry. Proc. R. Soc. A.

[43] Fürstner A., Leitner A. (2002). Iron-Catalyzed Cross-Coupling Reactions of Alkyl-Grignard Reagents with Aryl Chlorides, Tosylates, and Triflates. Angew. Chem. Int. Ed..

[44] Fürstner A., Leitner A., Mendez M., Krause H. (2002). Iron-Catalyzed Cross-Coupling Reactions. J. Am. Chem. Soc..

[45] Czaplik W.M., Mayer M., Jacobi von Wangelin A. (2009). Domino Iron Catalysis: Direct Aryl-Alkyl Cross-Coupling. Angew. Chem. Int. Ed..

[46] Gülak S., Jacobi von Wangelin A. (2012). Chlorostyrenes in Iron-Catalyzed Biaryl Coupling Reactions. Angew. Chem. Int. Ed..

[47] Kuzmina O.M., Steib A.K., Markiewicz J.T., Flubacher D., Knochel P. (2013). Ligand-Accelerated Iron- and Cobalt-Catalyzed Cross-Coupling Reactions between N-Heteroaryl Halides and Aryl Magnesium Reagents. Angew. Chem. Int. Ed..

[48] Fürstner A., Martin R., Krause H., Seidel G., Goddard R., Lehmann C.W. (2008). Preparation, Structure, and Reactivity of Nonstabilized Organoiron Compounds. Implications for Iron-Catalyzed Cross Coupling Reactions. J. Am. Chem. Soc..

[49] Cassani C., Bergonzini G., Wallentin C.J. (2016). Active Species and Mechanistic Pathways in Iron-Catalyzed C–C Bond-Forming Cross-Coupling Reactions. ACS Catal..

[50] Gilman H., Beaber N.J., Meyers C.H. (1925). The Reaction Between Aryl Sulfonates and Organomagnesium Halides. J. Am. Chem. Soc..

[51] Liebman J., Greenberg A. (1974). The Origin of Rotational Barriers in Amides and Esters. Biophys. Chem..

[52] Piontek A., Szostak M. (2017). Iron-Catalyzed C(sp^2^)-C(sp^3^) Cross-Coupling of Alkyl Grignard Reagents with Polyaromatic Tosylates. Eur. J. Org. Chem..

[53] Agrawal T., Cook S.P. (2013). Iron-Catalyzed Cross-Coupling Reactions of Alkyl Grignards with Aryl Sulfamates and Tosylates. Org. Lett..

[54] Bisz E., Kardela M., Szostak M. (2019). Ligand Effect on Iron-Catalyzed Cross-Coupling Reactions: Evaluation of Amides as O-coordinating Ligands. ChemCatChem.

[55] Ratushnyy M., Kamenova M., Gevorgyan V. (2018). A Mild Light-Induced Cleavage of S–O Bond of Aryl Sulfonate Esters Enables Efficient Sulfonylation of Vinylarenes. Chem. Sci..

[56] Perez K.A., Rogers C.R., Weiss E.A. (2020). Quantum Dot-Catalyzed Photoreductive Removal of Sulfonyl-Based Protecting Groups. Angew. Chem. Int. Ed..

