# Supplementary material for: Iron-Catalyzed Cross-Coupling Reactions of Alkyl Grignards with Aryl Chlorobenzenesulfonates
Source: Molecules. 2021 Sep 29;26(19):5895. doi: 10.3390/molecules26195895 (PMC8510395; doi:10.3390/molecules26195895)
Supplement: Supplementary file 1 [file molecules-26-05895-s001.zip › molecules-1390951-supplementary.pdf]

Iron-Catalyzed Cross-Coupling Reactions of Alkyl Grignards with Aryl Chlorobenzenesulfonates

Elwira Bisz*,†

†*Department of Chemistry, Opole University, 48 Oleska Street, 45-052 Opole, Poland*

ebisz@uni.opole.pl

Supporting Information

Table of Contents

Table of Contents SI-1
^{1}H and ^{13}C NMR Spectra SI-2

Corresponding Author:

Dr. E. Bisz
Department of Chemistry, Opole University
48 Oleska Street, 45-052 Opole, Poland
E-mail: ebisz@uni.opole.pl

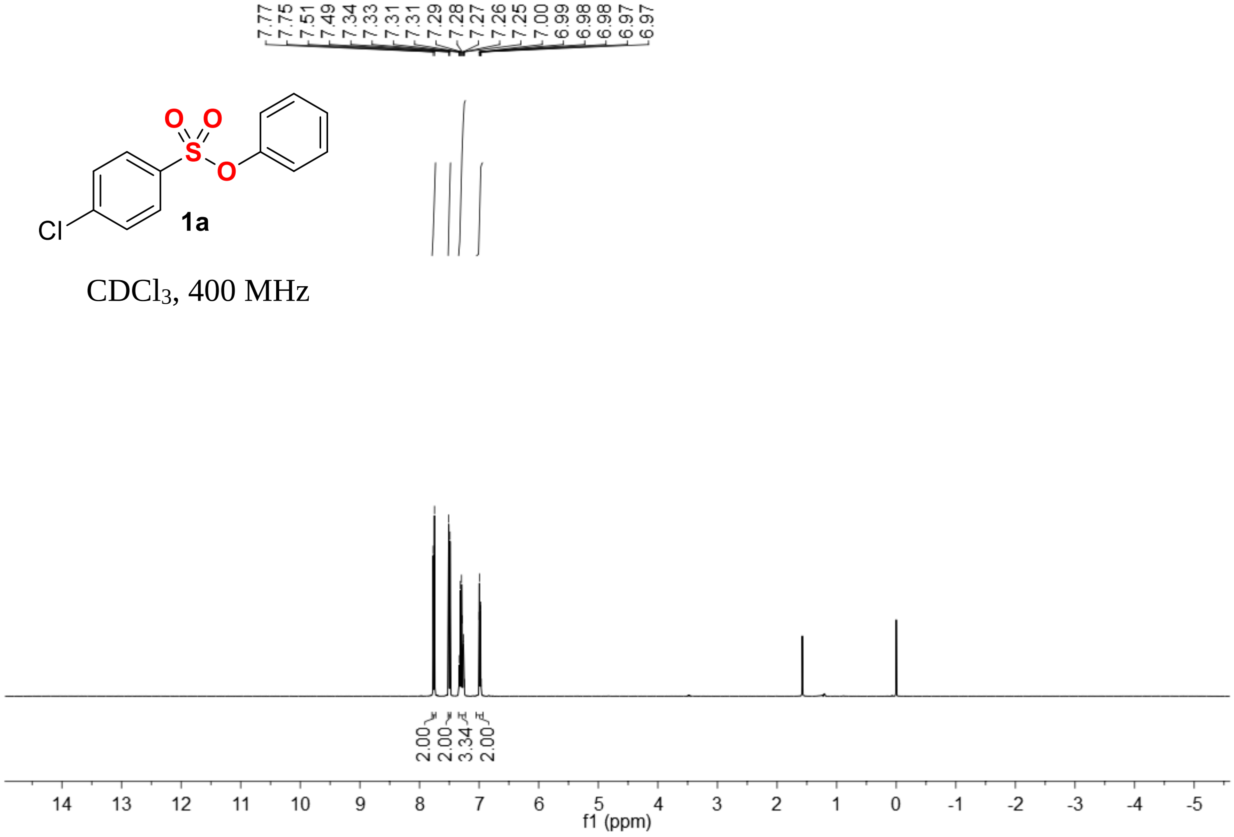

$CDCl_3$, 400 MHz

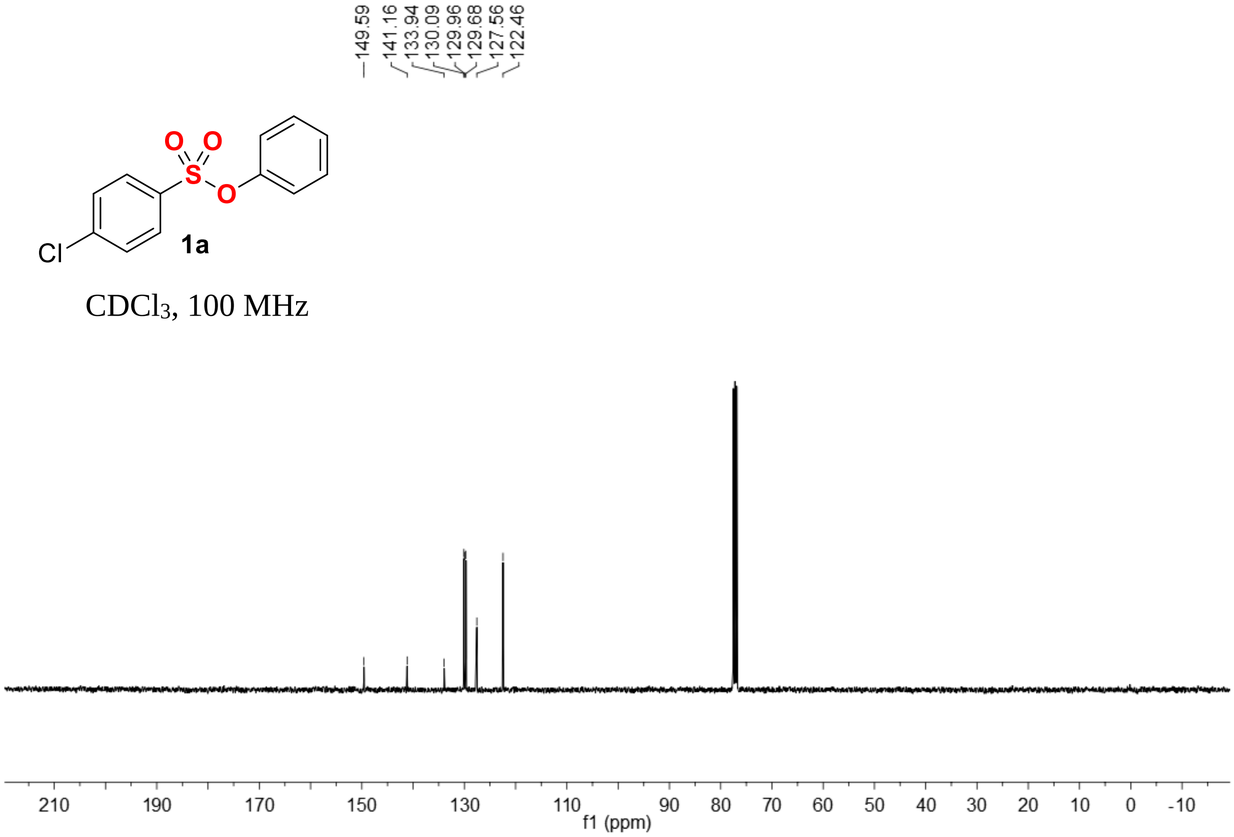

$CDCl_3$, 100 MHz

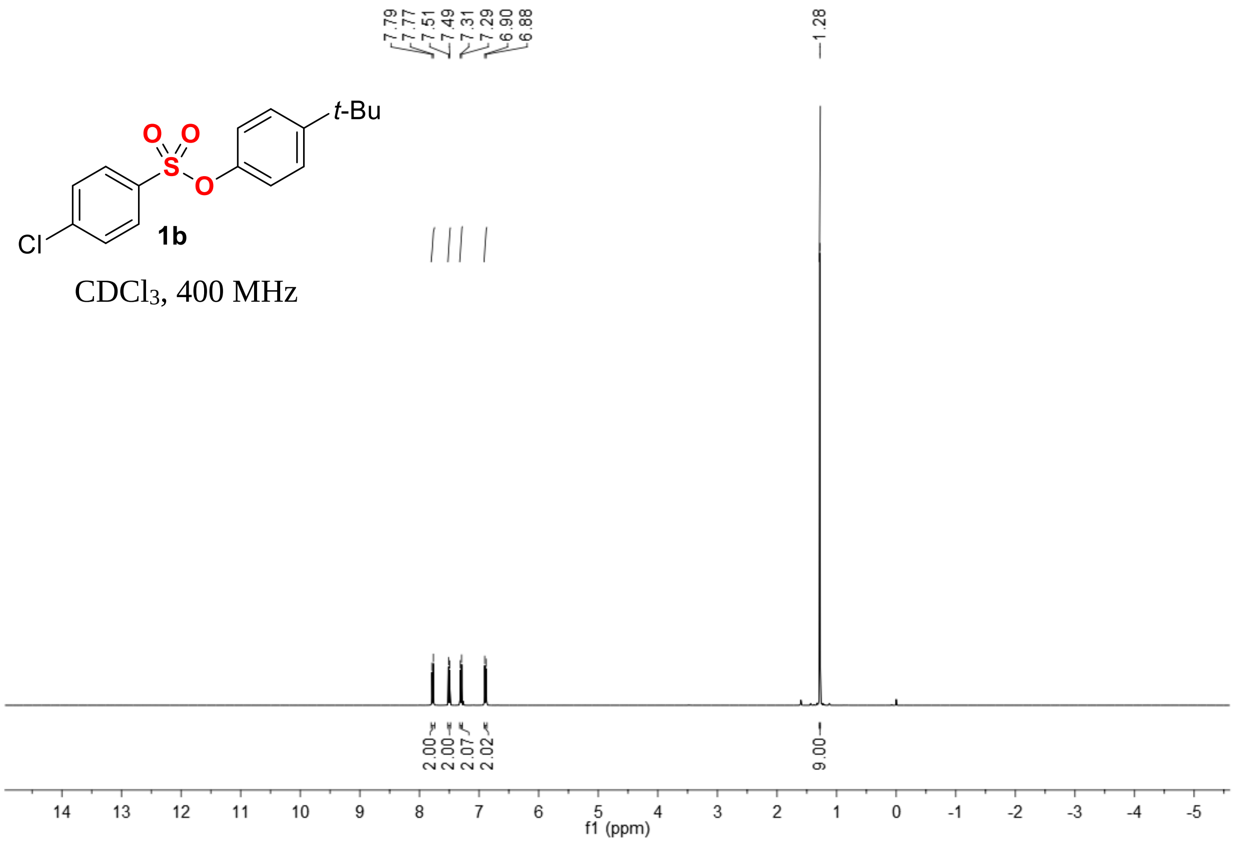
7.79
7.77
7.51
7.49
7.31
7.29
6.90
6.88
1.28
t-Bu
O O
S
O
Cl
1b
CDCl3, 400 MHz
2.00
2.00
2.07
2.02
9.00
f1 (ppm)

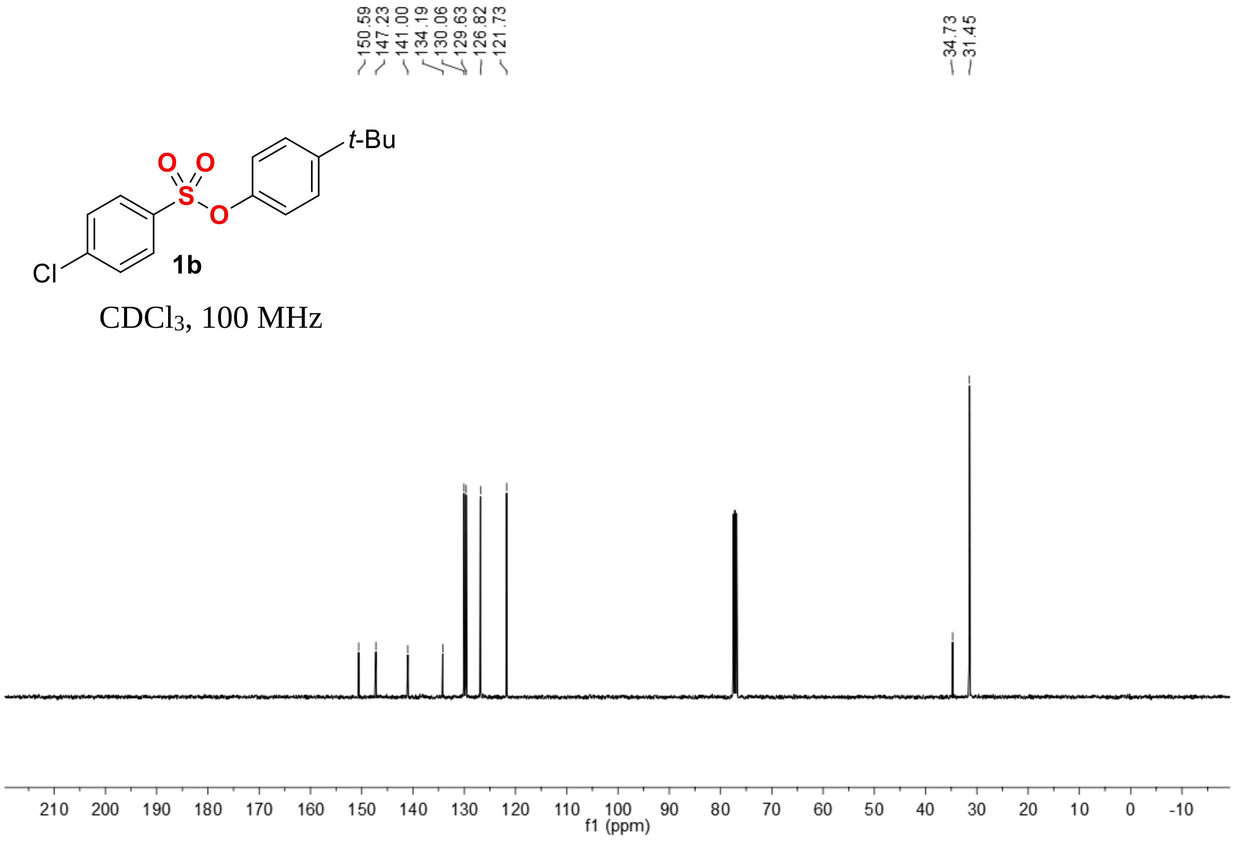
150.59
147.23
141.00
134.19
130.06
129.63
126.82
121.73
34.73
31.45
t-Bu
O O
S
O
Cl
1b
CDCl3, 100 MHz
f1 (ppm)

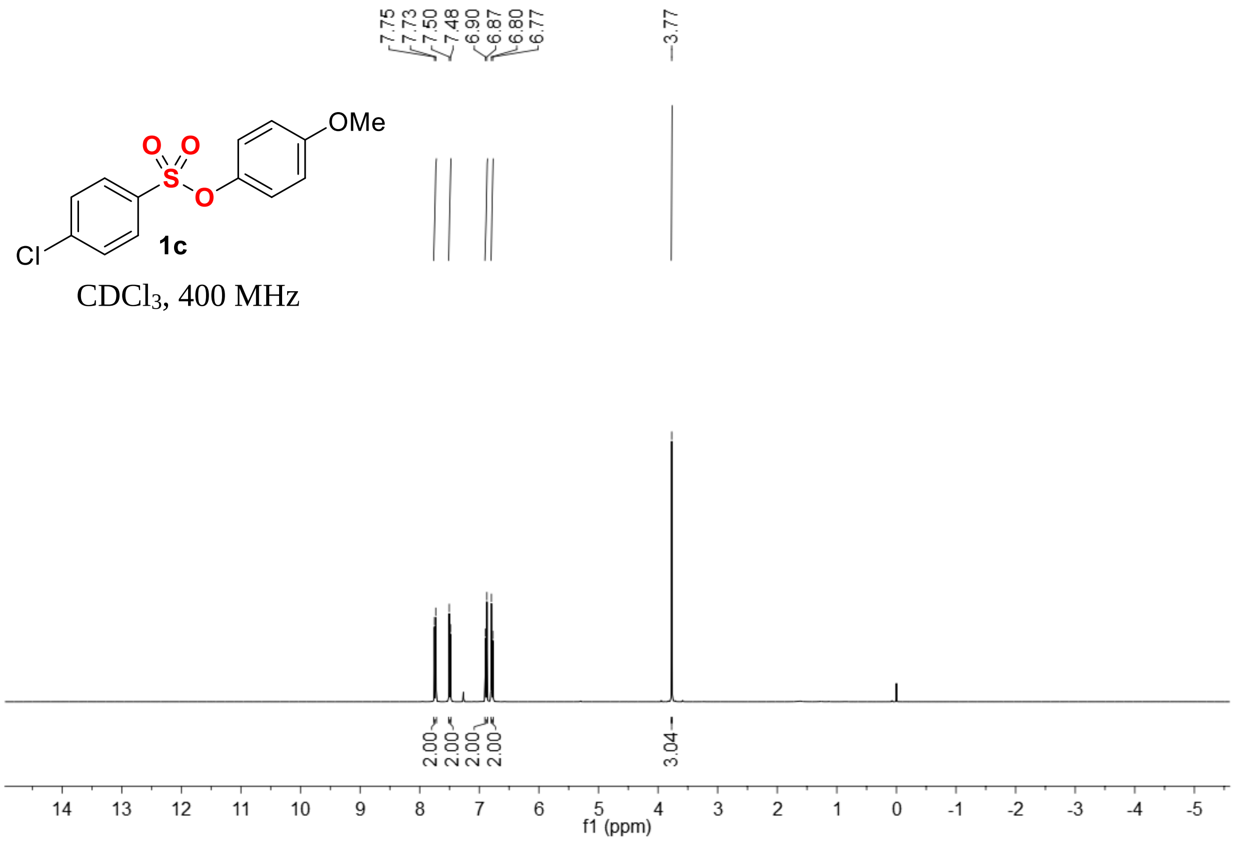
7.75
7.73
7.50
7.48
6.90
6.87
6.80
6.77
3.77
OMe
Cl
1c
CDCl3, 400 MHz
2.00
2.00
2.00
2.00
3.04
f1 (ppm)

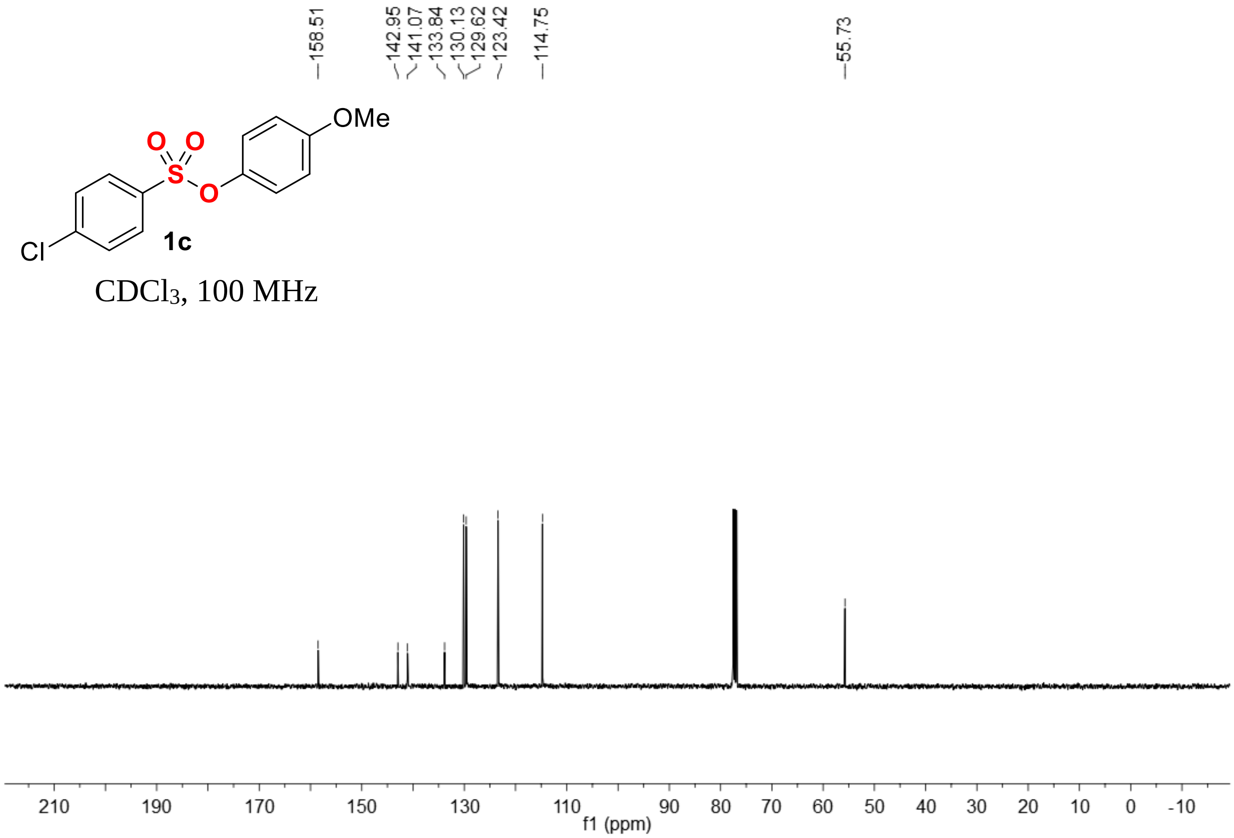
158.51
142.95
141.07
133.84
130.13
129.62
123.42
114.75
55.73
OMe
Cl
1c
CDCl3, 100 MHz
f1 (ppm)

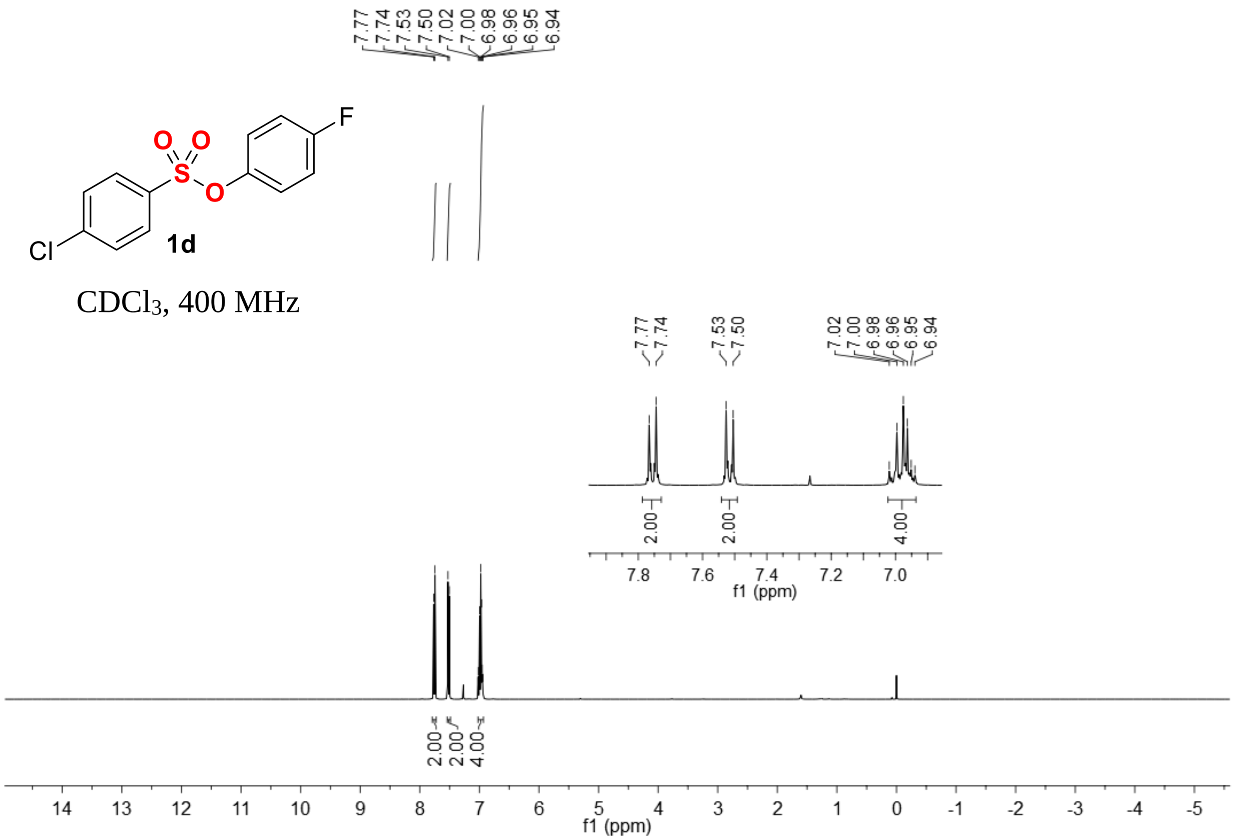
7.77
7.74
7.53
7.50
7.02
7.00
6.98
6.96
6.95
6.94
1d
CDCl3, 400 MHz
2.00
2.00
4.00
f1 (ppm)

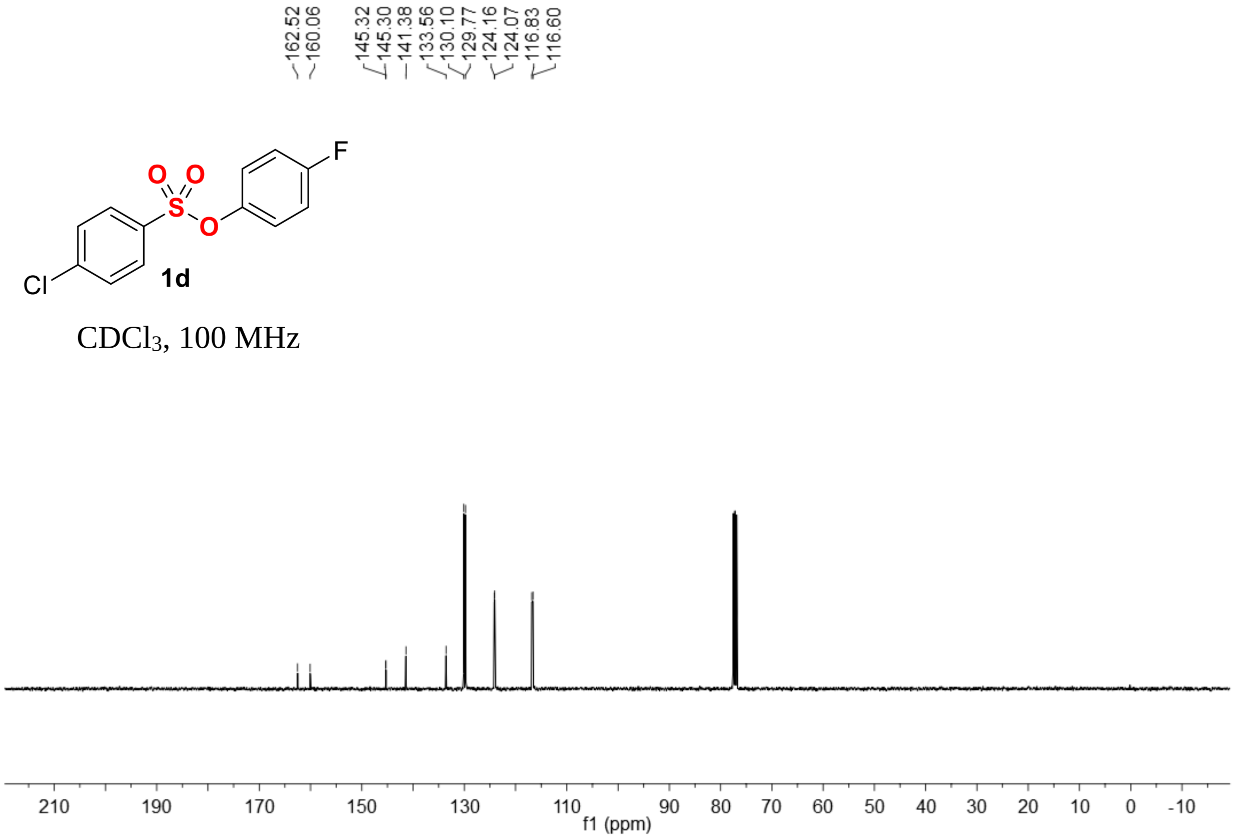
162.52
160.06
145.32
145.30
141.38
133.56
130.10
129.77
124.16
124.07
116.83
116.60
1d
CDCl3, 100 MHz
f1 (ppm)

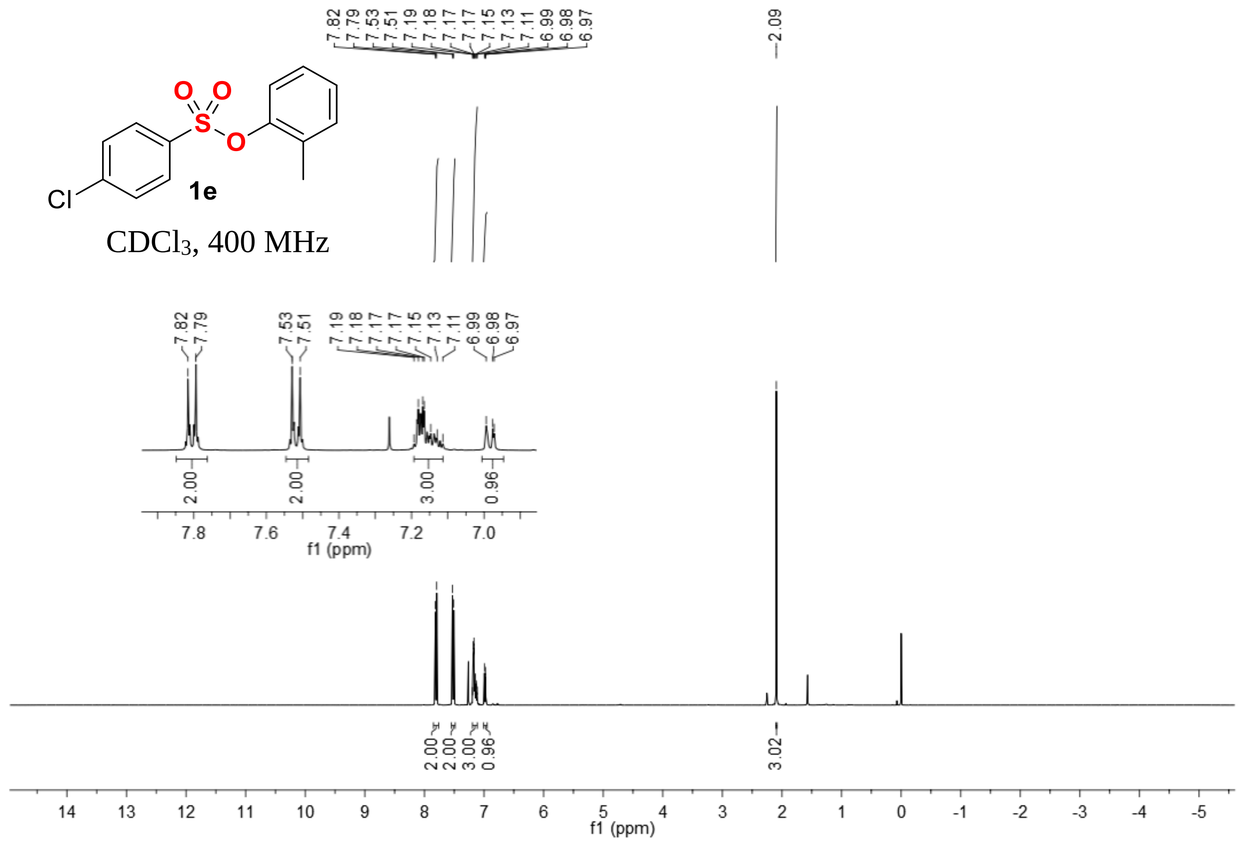
7.82
7.79
7.53
7.51
7.19
7.18
7.17
7.17
7.15
7.13
7.11
6.99
6.98
6.97
2.09
O
O
S
O
Cl
1e
CDCl3, 400 MHz
2.00
2.00
3.00
0.96
7.8 7.6 7.4 7.2 7.0
f1 (ppm)
3.02
14 13 12 11 10 9 8 7 6 5 4 3 2 1 0 -1 -2 -3 -4 -5
f1 (ppm)

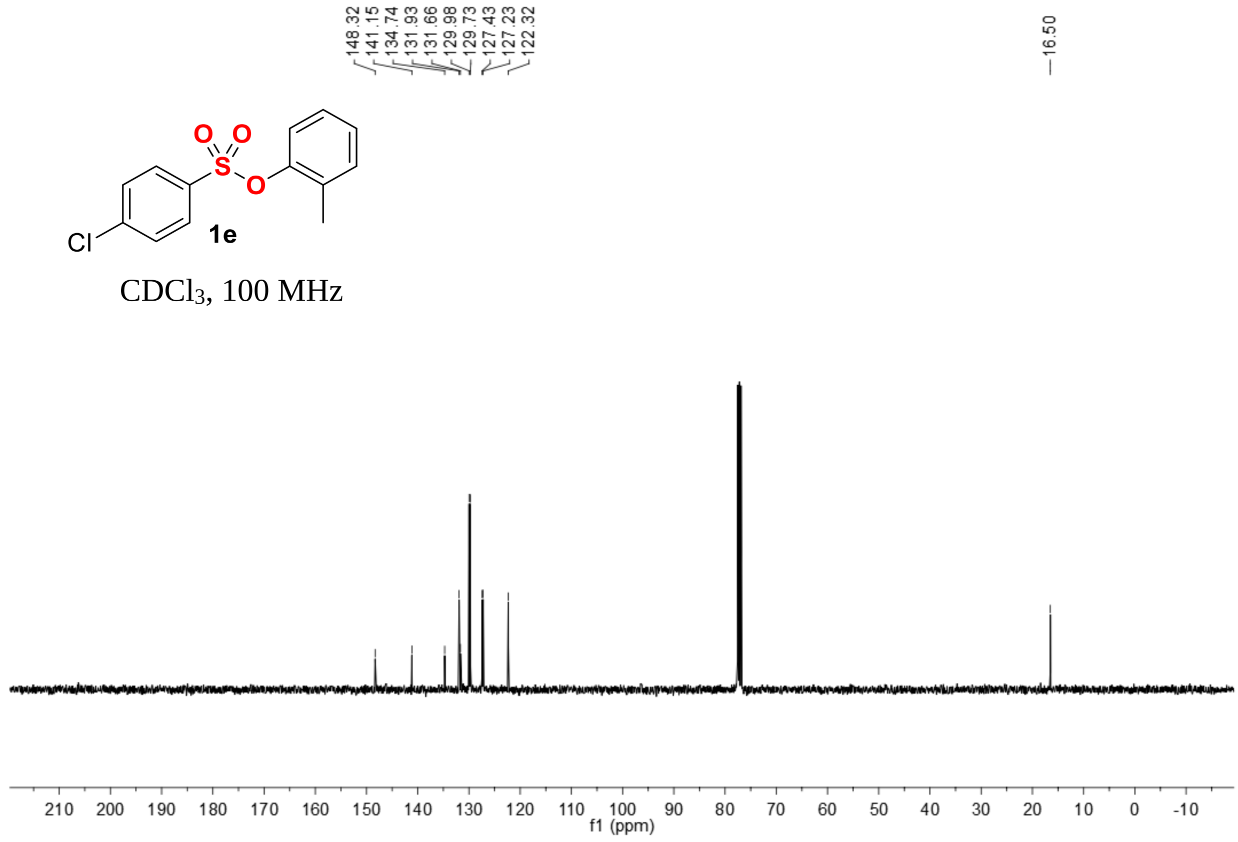
148.32
141.15
134.74
131.93
131.66
129.98
129.73
127.43
127.23
122.32
16.50
O
O
S
O
Cl
1e
CDCl3, 100 MHz
210 200 190 180 170 160 150 140 130 120 110 100 90 80 70 60 50 40 30 20 10 0 -10
f1 (ppm)

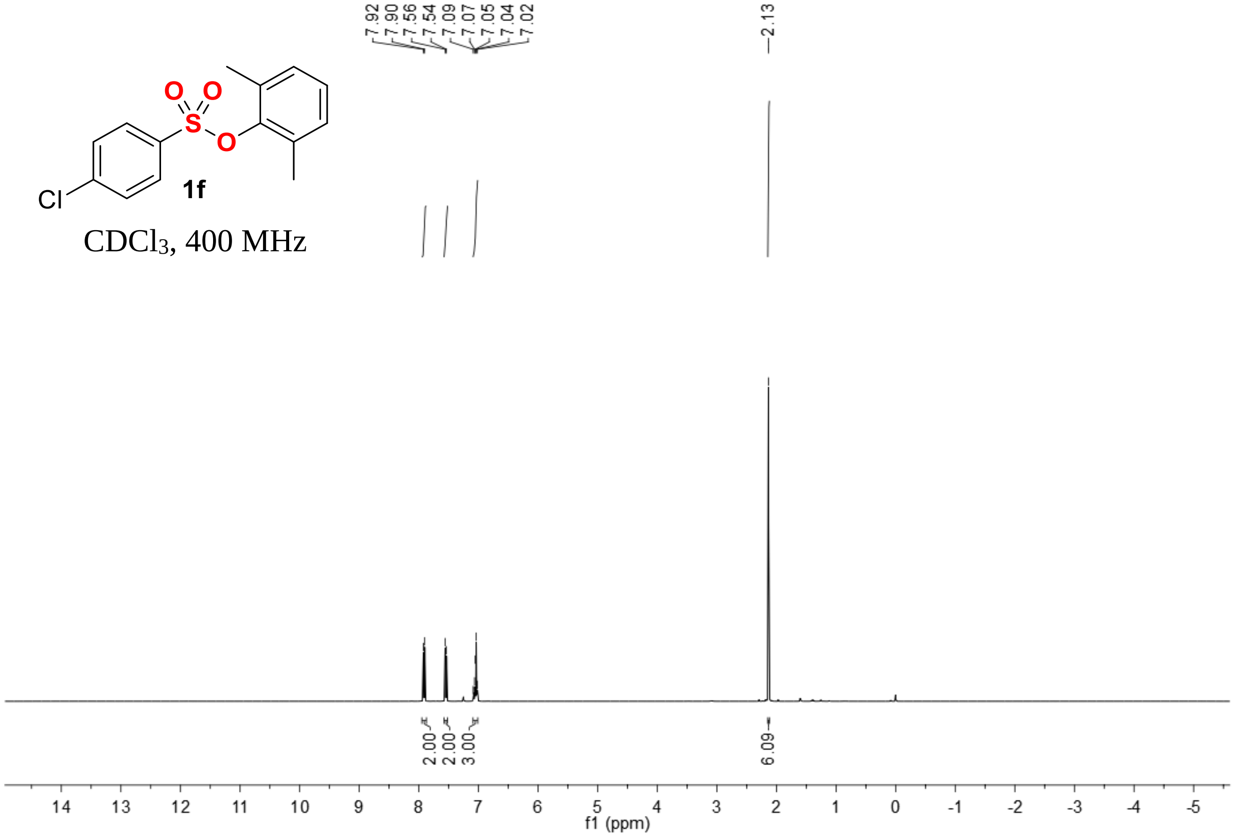
7.92
7.90
7.56
7.54
7.09
7.07
7.05
7.04
7.02
2.13
O
O
S
O
Cl
1f
CDCl3, 400 MHz
2.00
2.00
3.00
6.09
14 13 12 11 10 9 8 7 6 5 4 3 2 1 0 -1 -2 -3 -4 -5
f1 (ppm)

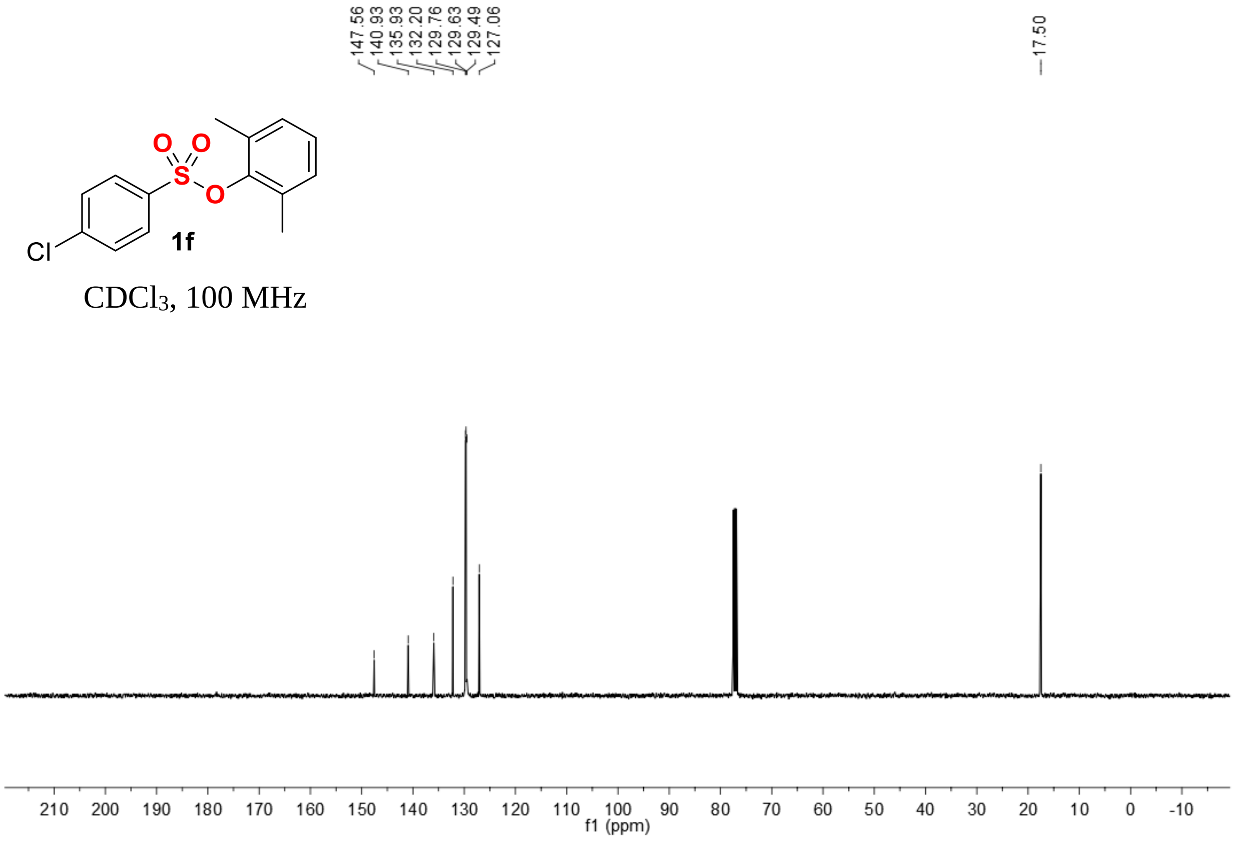
147.56
140.93
135.93
132.20
129.76
129.63
129.49
127.06
17.50
O
O
S
O
Cl
1f
CDCl3, 100 MHz
210 200 190 180 170 160 150 140 130 120 110 100 90 80 70 60 50 40 30 20 10 0 -10
f1 (ppm)

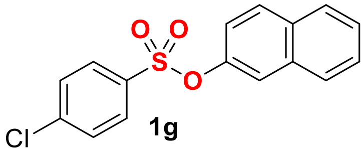

CDCl$_3$, 400 MHz

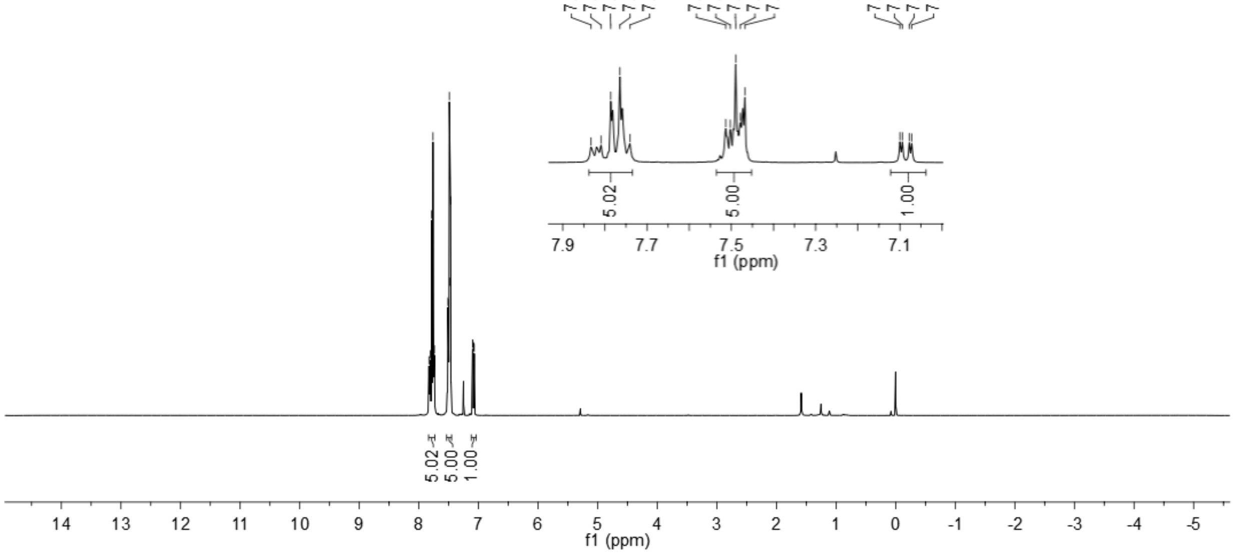

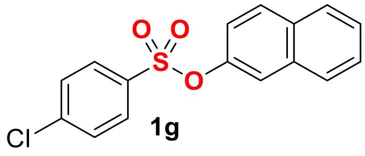

CDCl$_3$, 100 MHz

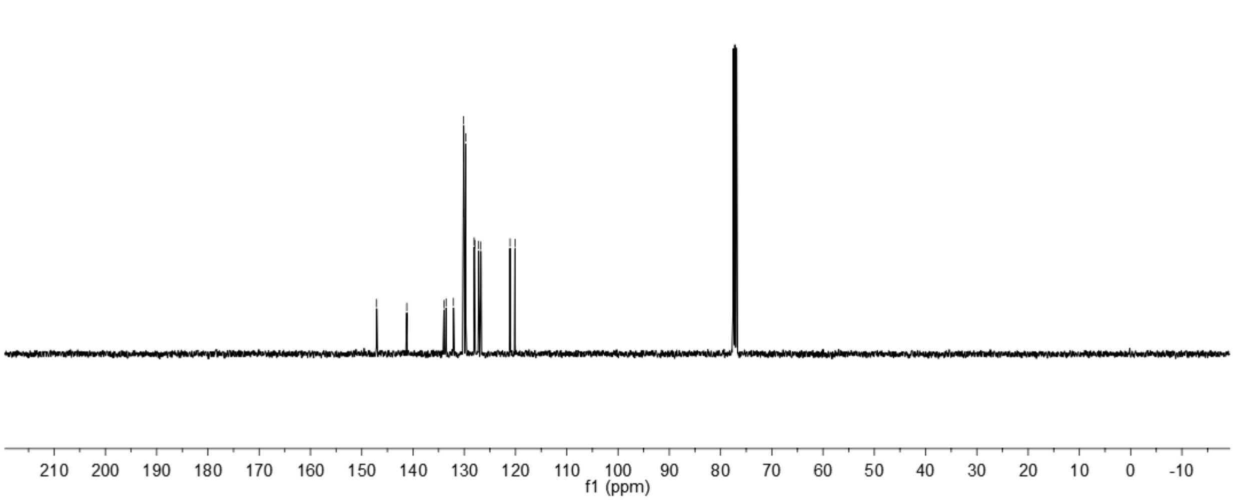

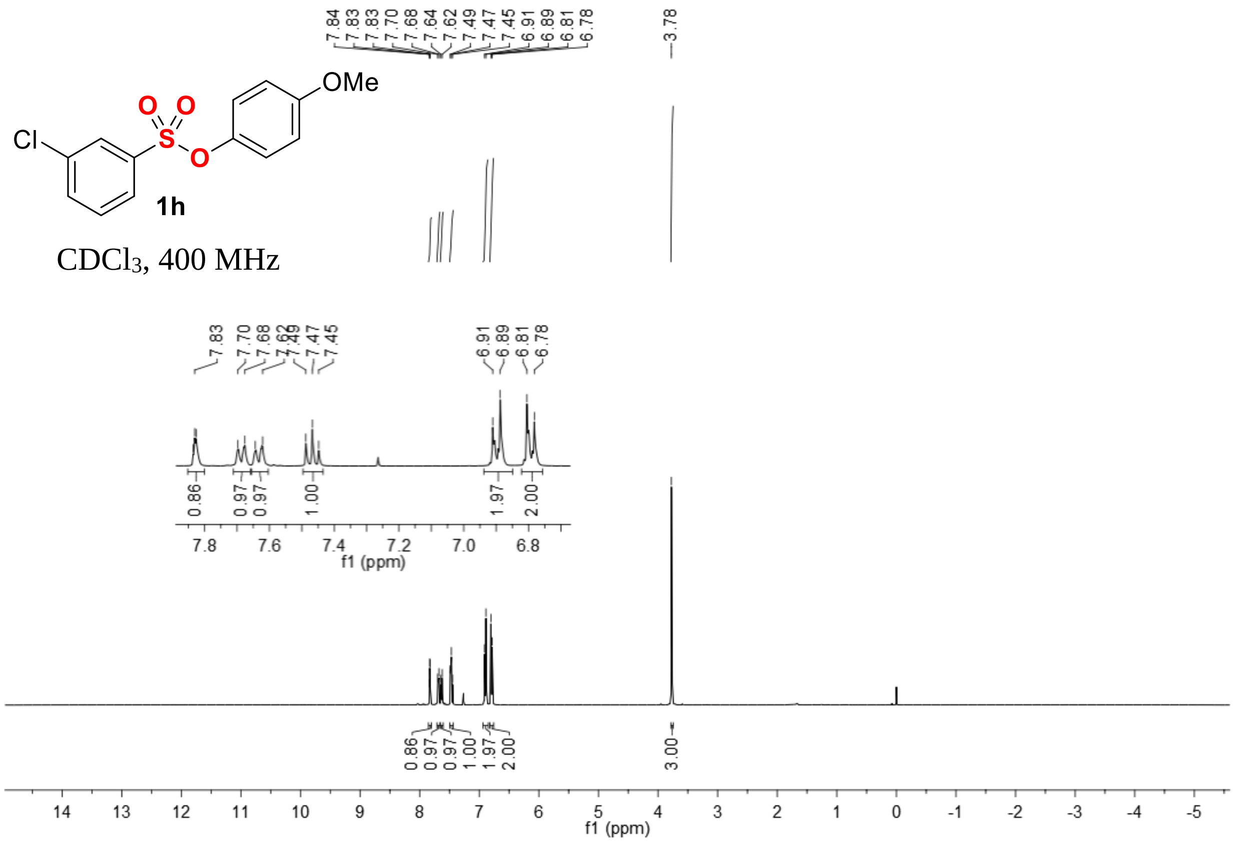
7.84 7.83 7.83 7.70 7.68 7.64 7.62 7.49 7.47 7.45 6.91 6.89 6.81 6.78
3.78
Cl
O
O
S
O
OMe
1h
CDCl3, 400 MHz
0.86 0.97 0.97 1.00 1.97 2.00
3.00
f1 (ppm)

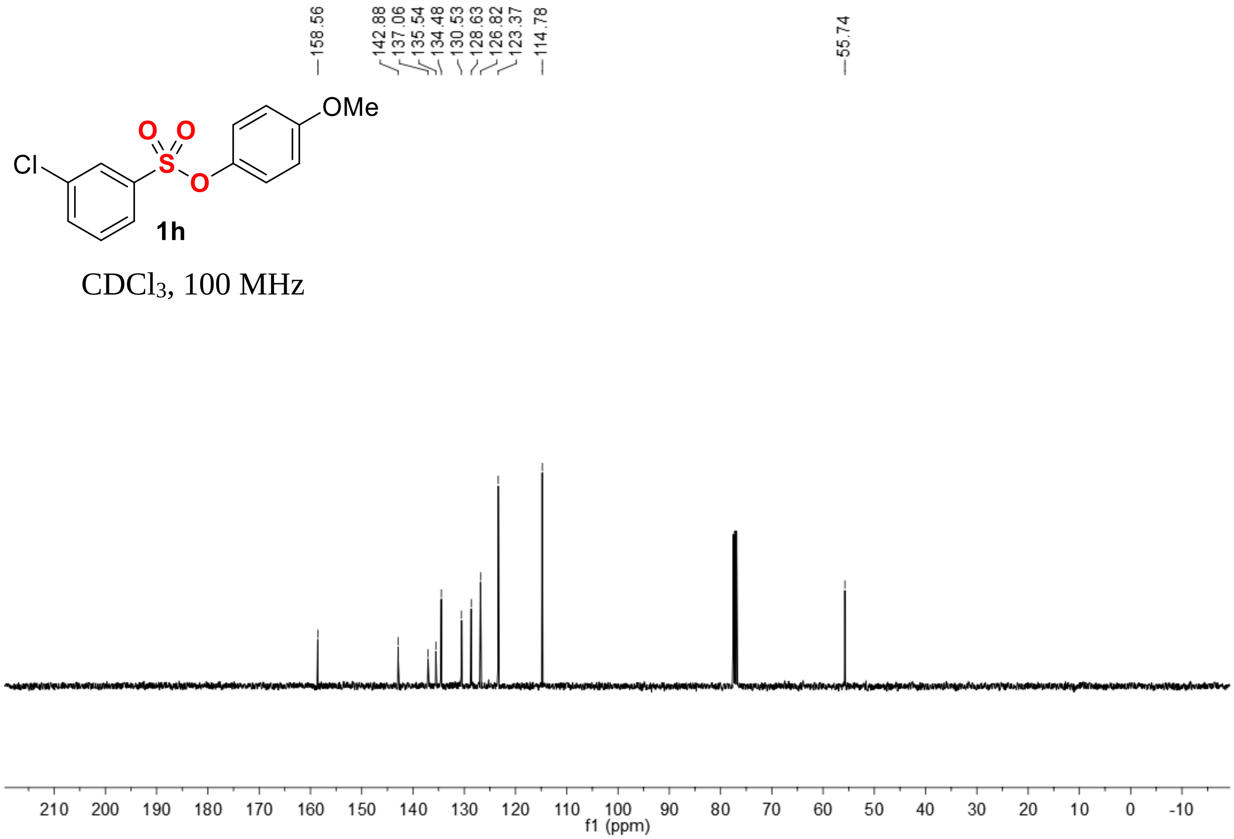
158.56
142.88 137.06 135.54 134.48 130.53 128.63 126.82 123.37 114.78
55.74
Cl
O
O
S
O
OMe
1h
CDCl3, 100 MHz
f1 (ppm)

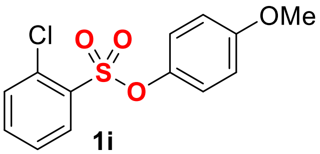

1i

CDCl$_3$, 400 MHz

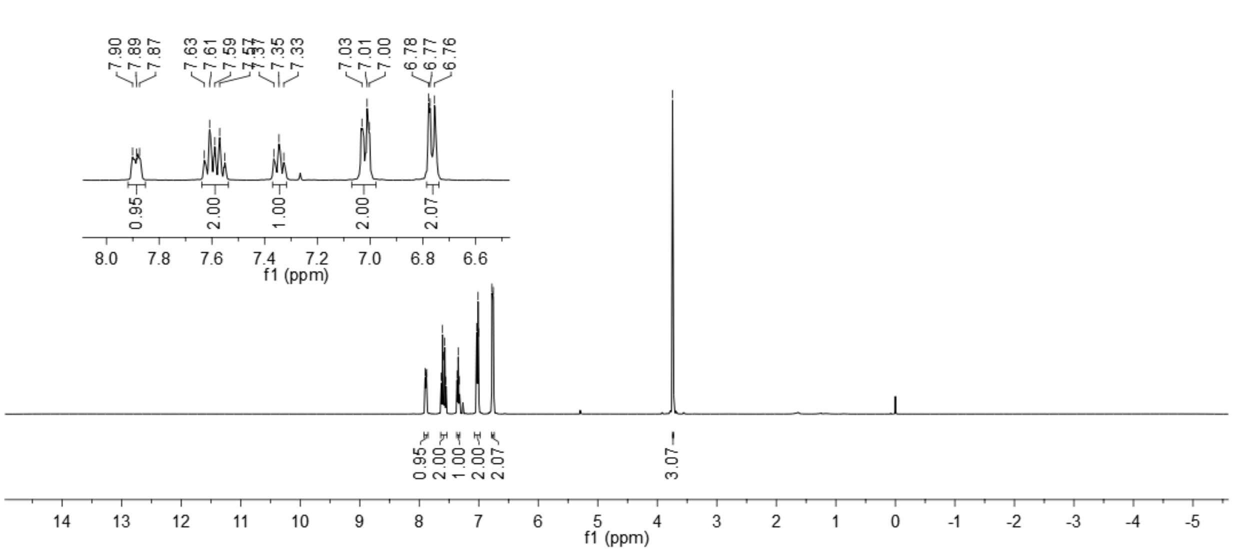

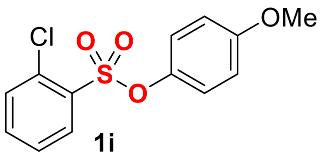

1i

CDCl$_3$, 100 MHz

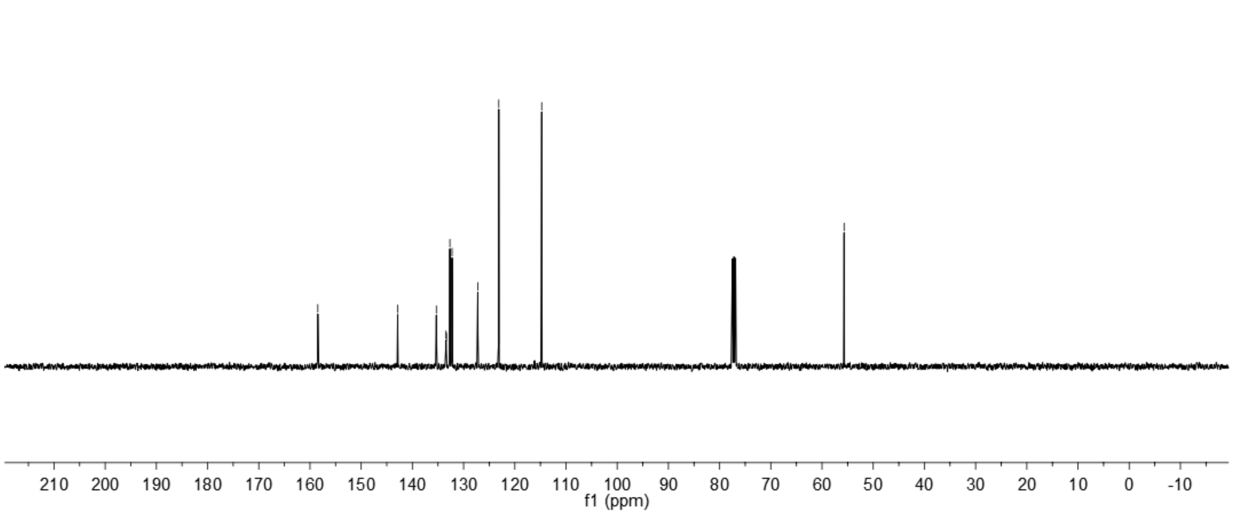

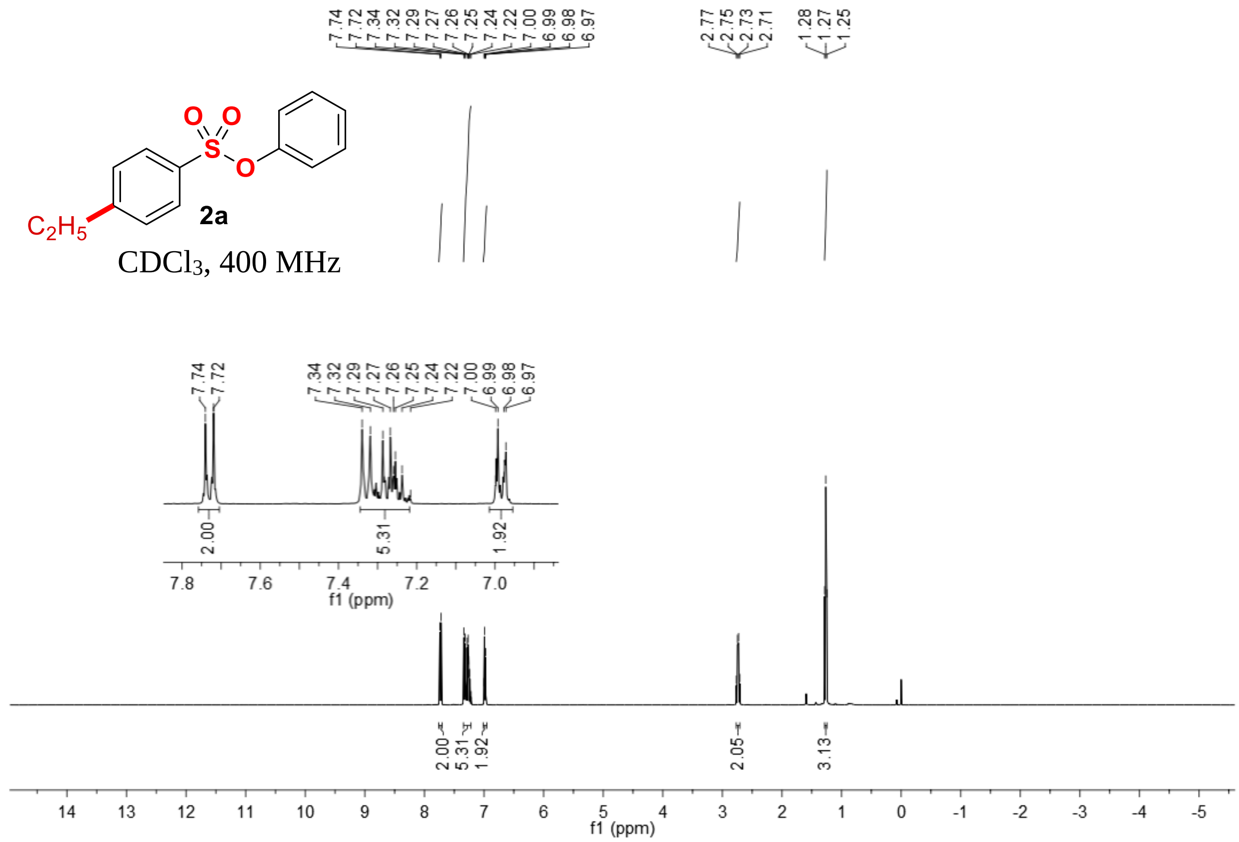

7.74 7.72 7.34 7.32 7.29 7.27 7.26 7.25 7.24 7.22 7.00 6.99 6.98 6.97
2.77 2.75 2.73 2.71
1.28 1.27 1.25
C_2H_5
2a
$CDCl_3$, 400 MHz
2.00
5.31
1.92
7.8 7.6 7.4 7.2 7.0
f1 (ppm)
2.00 5.31 1.92 2.05 3.13
14 13 12 11 10 9 8 7 6 5 4 3 2 1 0 -1 -2 -3 -4 -5
f1 (ppm)

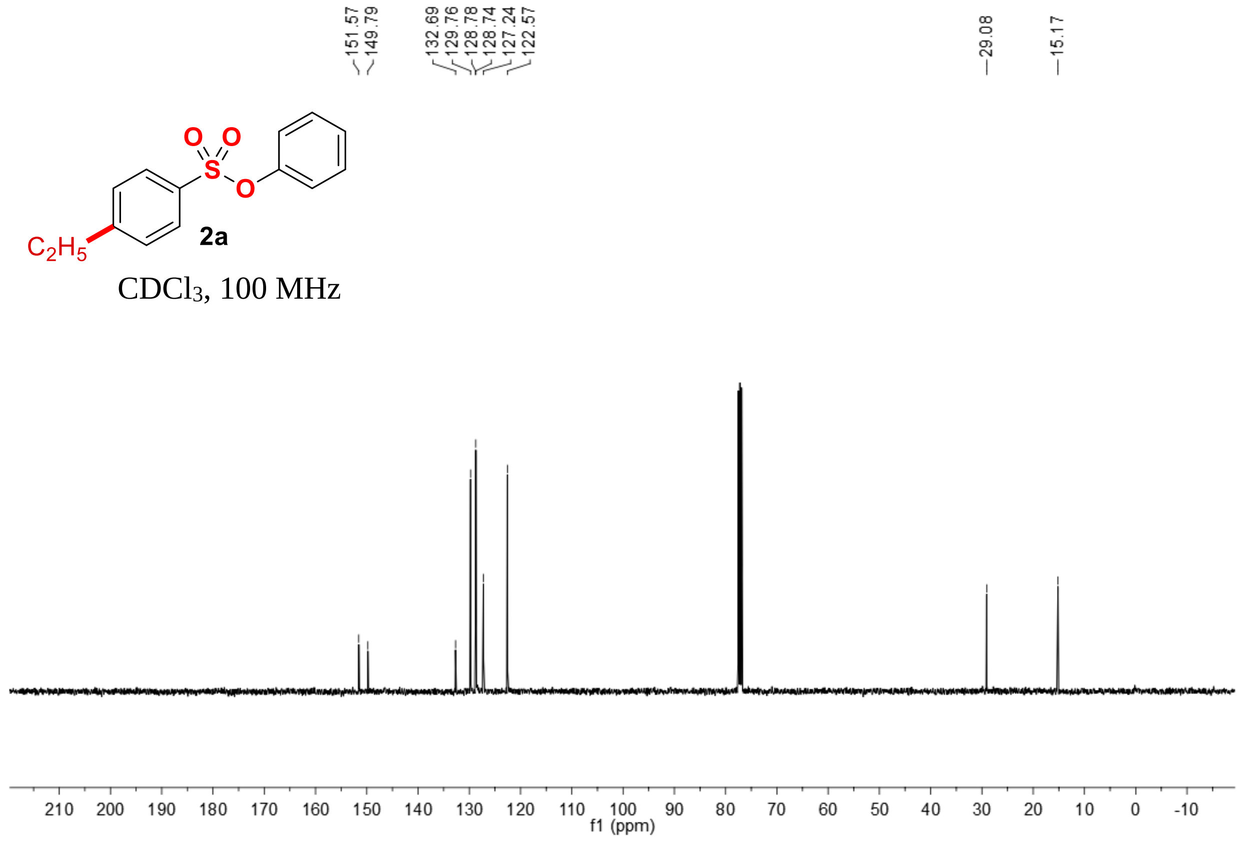

151.57 149.79
132.69 129.76 128.78 128.74 127.24 122.57
29.08
15.17
C_2H_5
2a
$CDCl_3$, 100 MHz
210 200 190 180 170 160 150 140 130 120 110 100 90 80 70 60 50 40 30 20 10 0 -10
f1 (ppm)

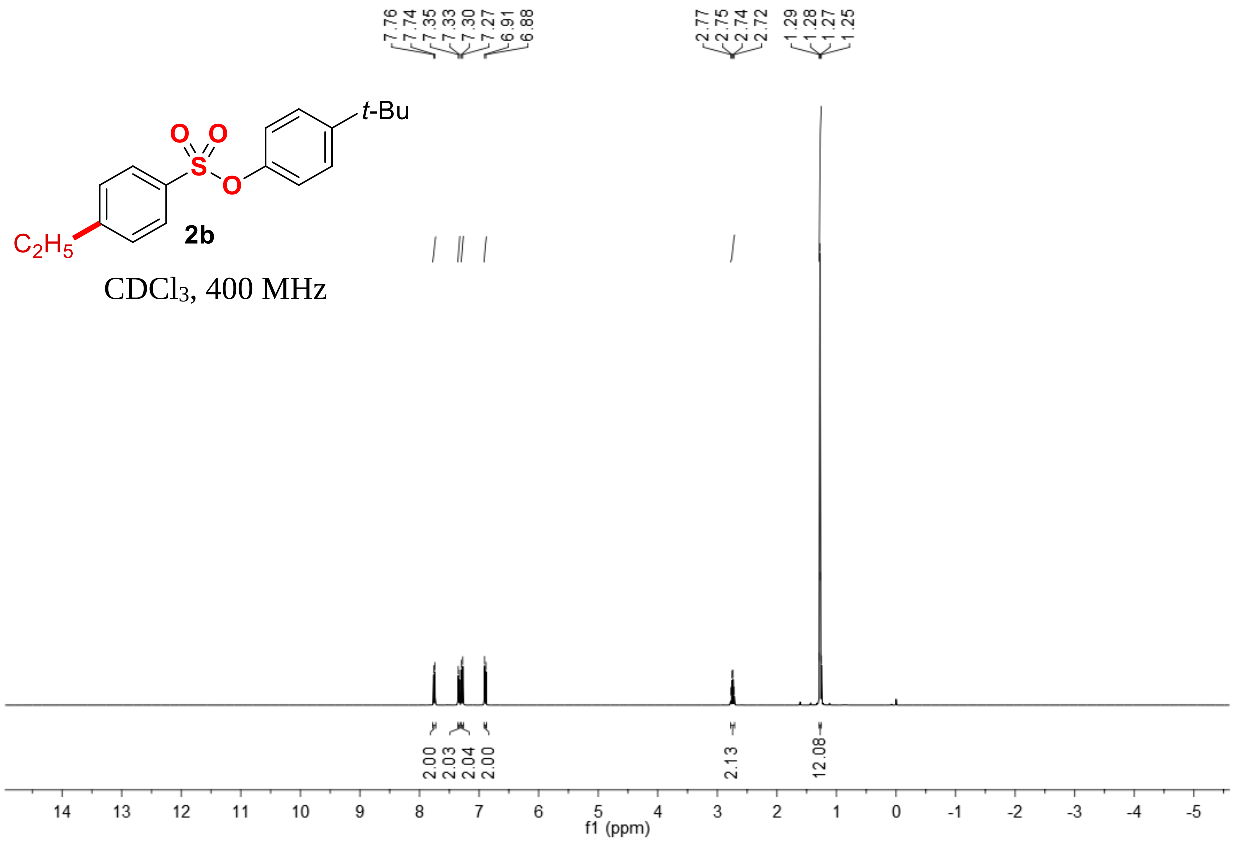
7.76
7.74
7.35
7.33
7.30
7.27
6.91
6.88
2.77
2.75
2.74
2.72
1.29
1.28
1.27
1.25
t-Bu
O O
S
O
C2H5
2b
CDCl3, 400 MHz
2.00
2.03
2.04
2.00
2.13
12.08
f1 (ppm)

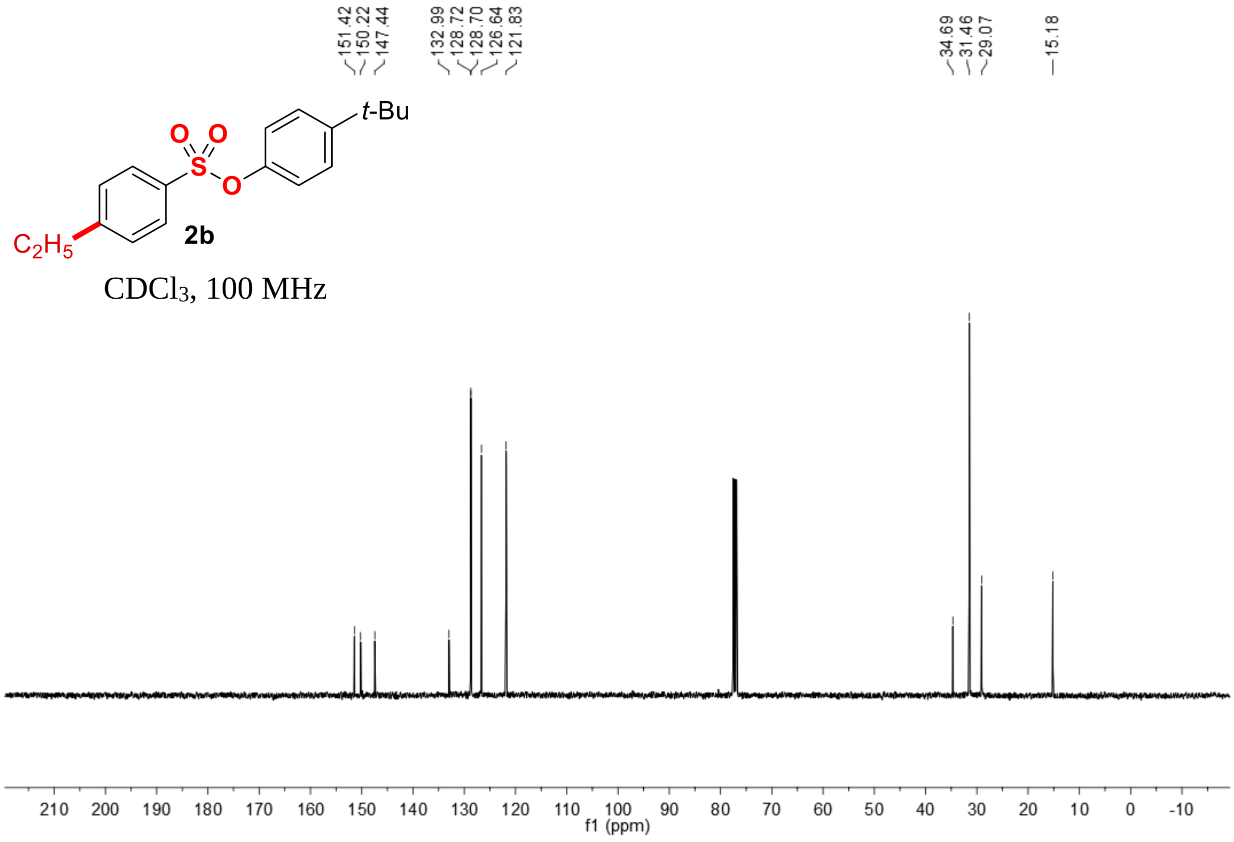
151.42
150.22
147.44
132.99
128.72
128.70
126.64
121.83
34.69
31.46
29.07
15.18
t-Bu
O O
S
O
C2H5
2b
CDCl3, 100 MHz
f1 (ppm)

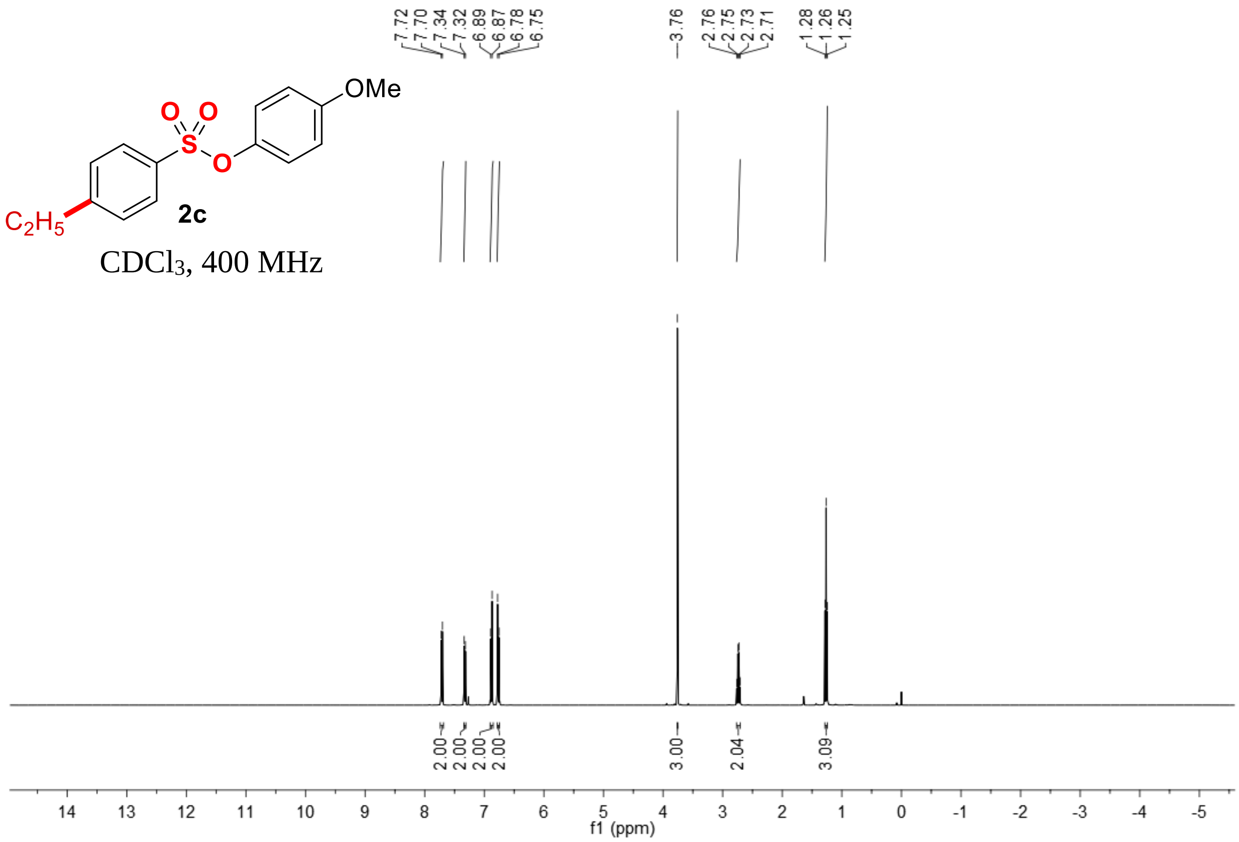
7.72
7.70
7.34
7.32
6.89
6.87
6.78
6.75
3.76
2.76
2.75
2.73
2.71
1.28
1.26
1.25
OMe
O
O
S
O
C_2H_5
2c
$CDCl_3$, 400 MHz
2.00
2.00
2.00
2.00
3.00
2.04
3.09
f1 (ppm)

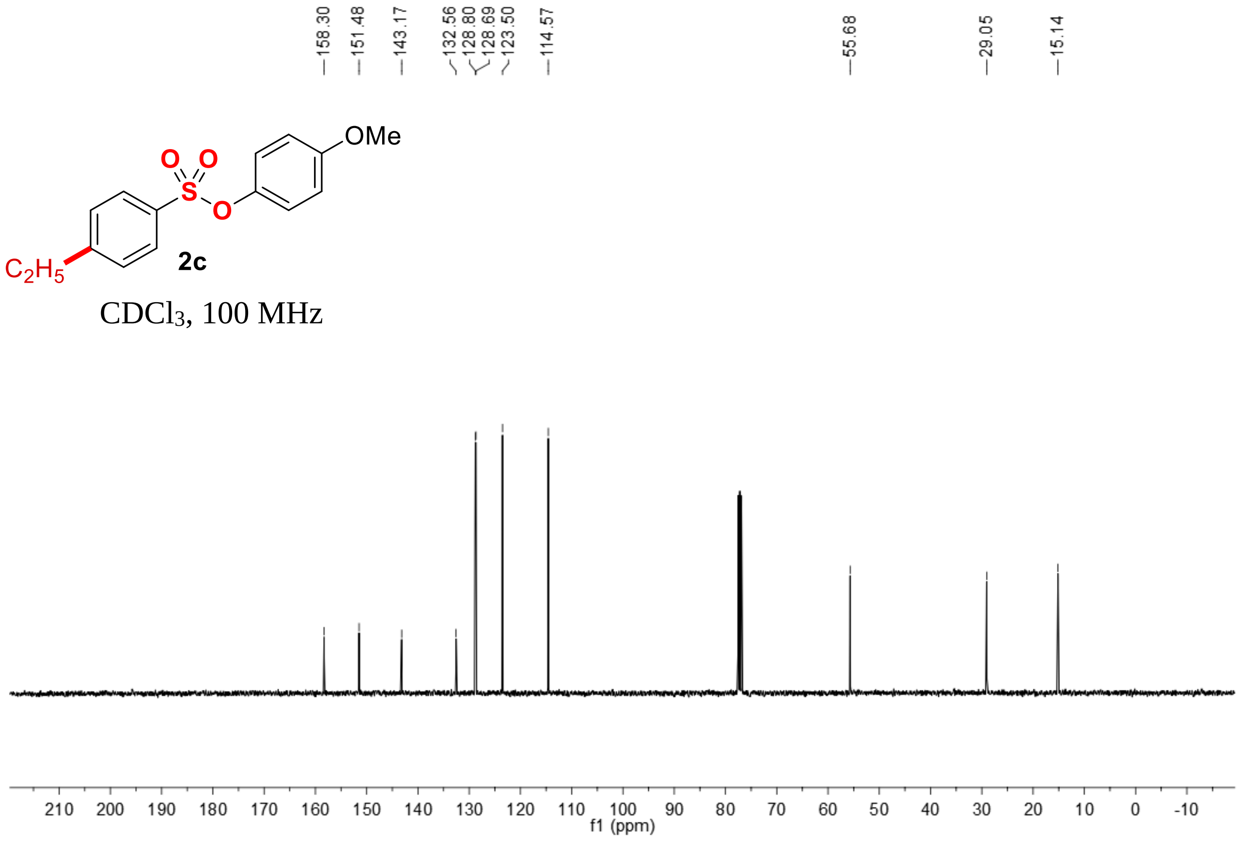
158.30
151.48
143.17
132.56
128.80
128.69
123.50
114.57
55.68
29.05
15.14
OMe
O
O
S
O
C_2H_5
2c
$CDCl_3$, 100 MHz
f1 (ppm)

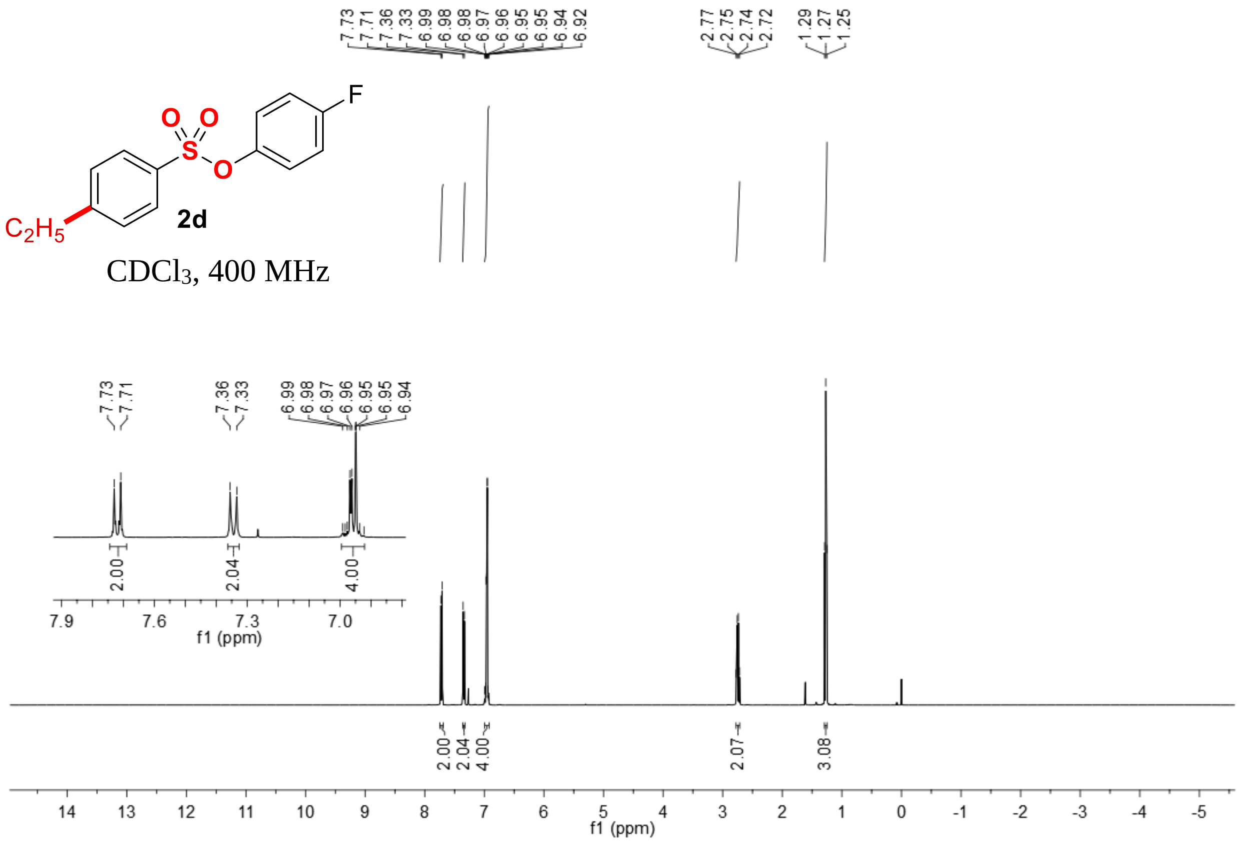
7.73
7.71
7.36
7.33
6.99
6.98
6.98
6.97
6.96
6.95
6.95
6.94
6.92
2.77
2.75
2.74
2.72
1.29
1.27
1.25
F
O
O
S
O
C2H5
2d
CDCl3, 400 MHz
2.00
2.04
4.00
2.07
3.08
f1 (ppm)

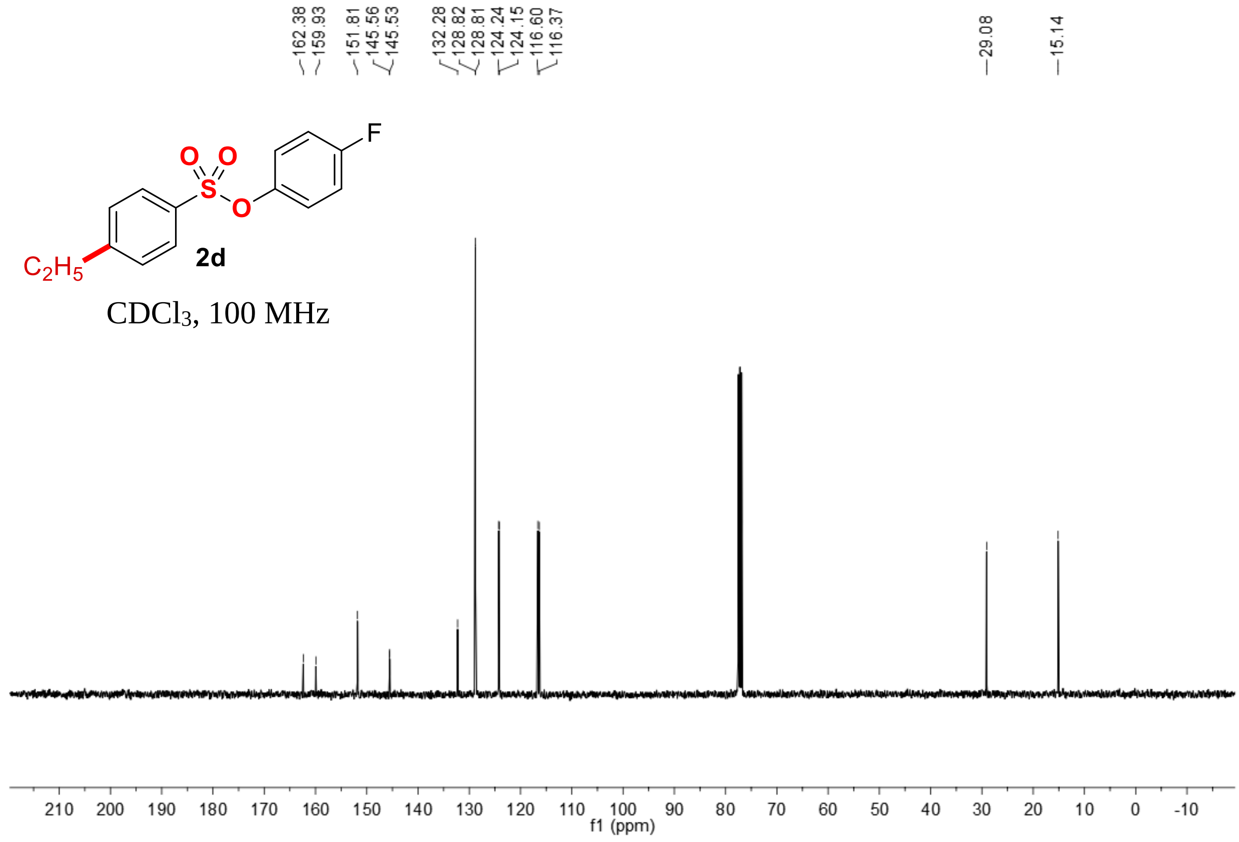
162.38
159.93
151.81
145.56
145.53
132.28
128.82
128.81
124.24
124.15
116.60
116.37
29.08
15.14
F
O
O
S
O
C2H5
2d
CDCl3, 100 MHz
f1 (ppm)

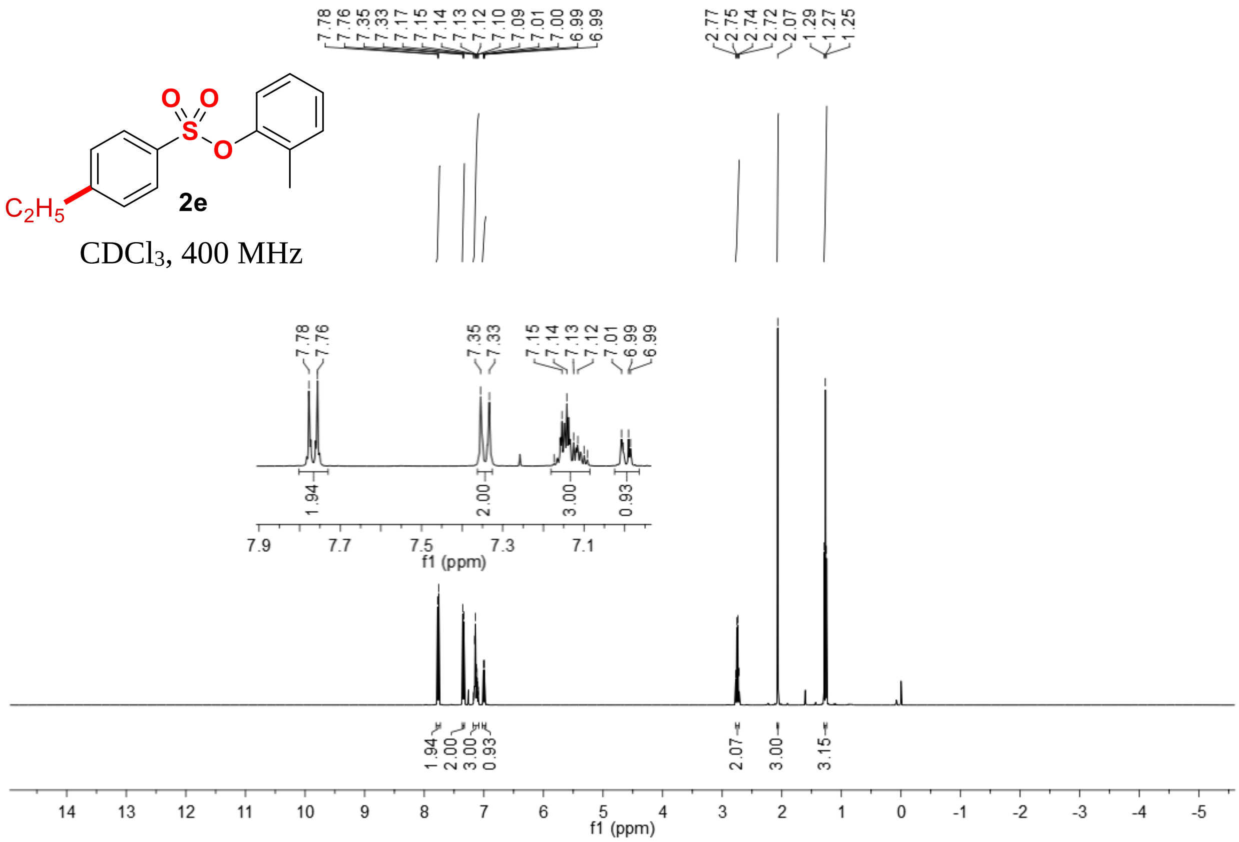
2e
CDCl3, 400 MHz
7.78
7.76
7.35
7.33
7.17
7.15
7.14
7.13
7.12
7.10
7.09
7.01
7.00
6.99
6.99
2.77
2.75
2.74
2.72
2.07
1.29
1.27
1.25
1.94
2.00
3.00
0.93
2.07
3.00
3.15
f1 (ppm)

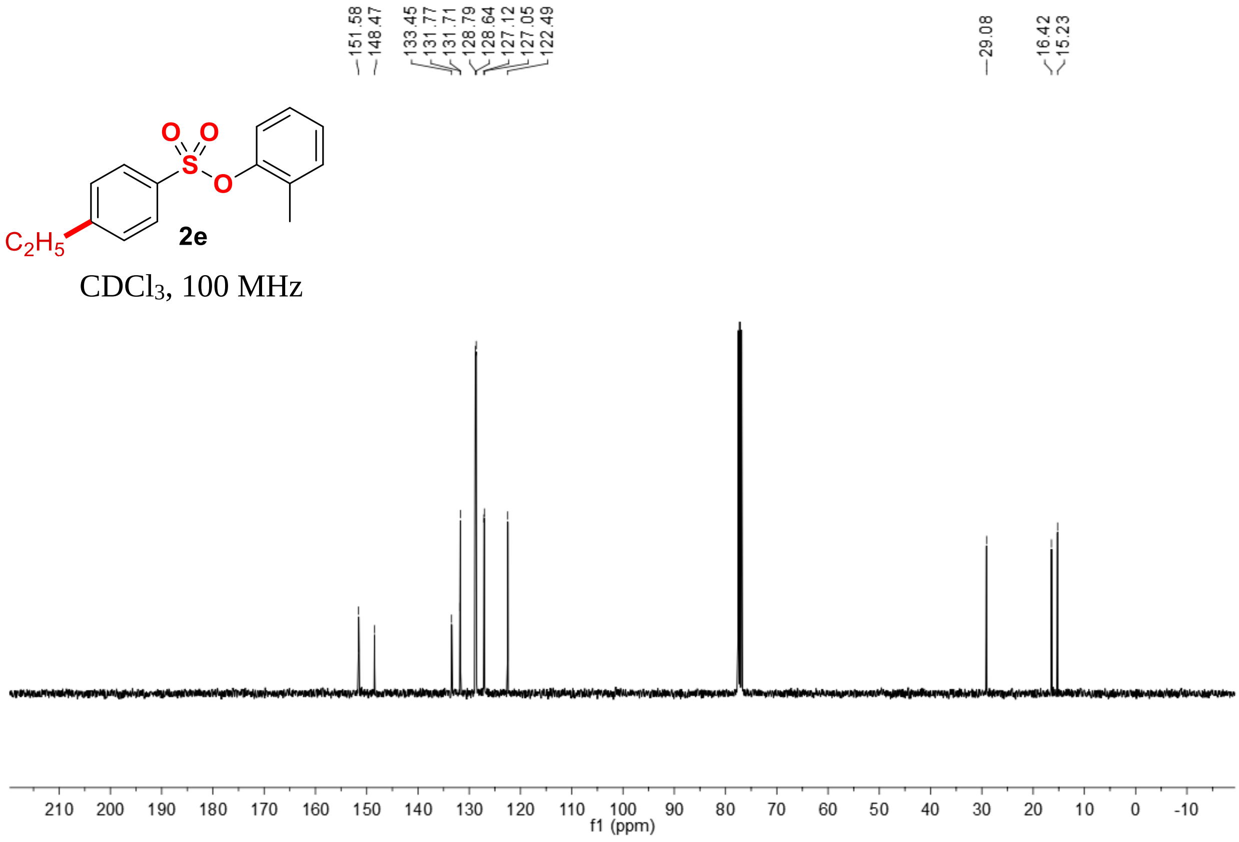
2e
CDCl3, 100 MHz
151.58
148.47
133.45
131.77
131.71
128.79
128.64
127.12
127.05
122.49
29.08
16.42
15.23
f1 (ppm)

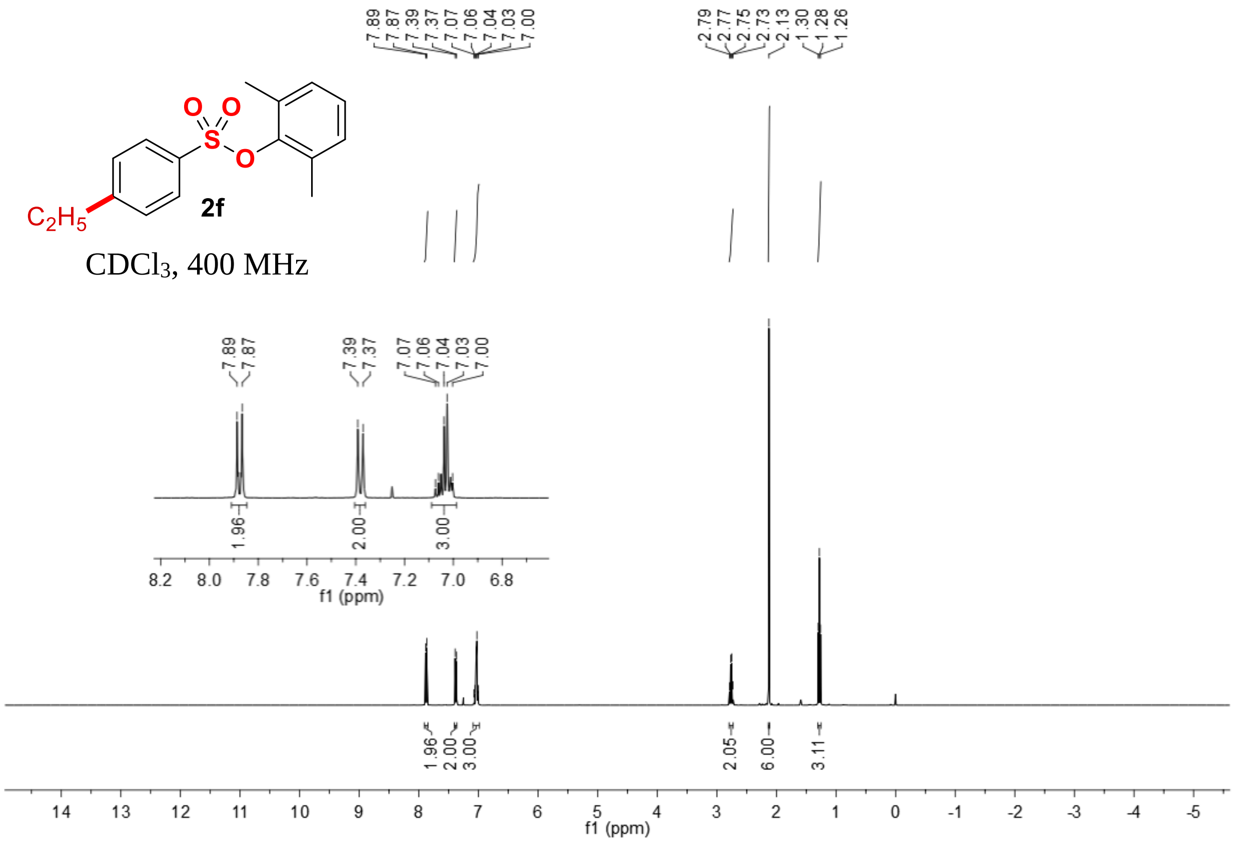

2f
CDCl3, 400 MHz
7.89 7.87 7.39 7.37 7.07 7.06 7.04 7.03 7.00
2.79 2.77 2.75 2.73 2.13 1.30 1.28 1.26
1.96 2.00 3.00
2.05 6.00 3.11
f1 (ppm)

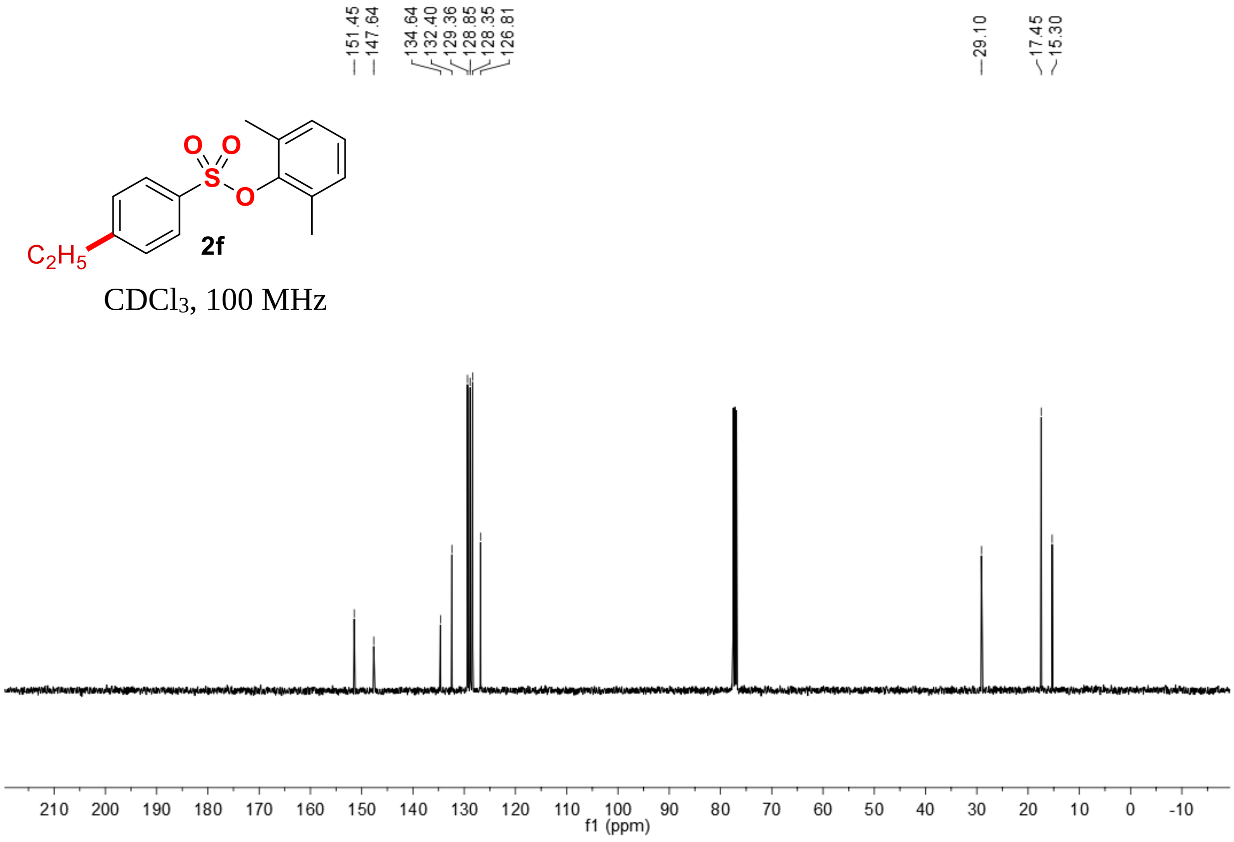

2f
CDCl3, 100 MHz
151.45 147.64 134.64 132.40 129.36 128.85 128.35 126.81
29.10 17.45 15.30
f1 (ppm)

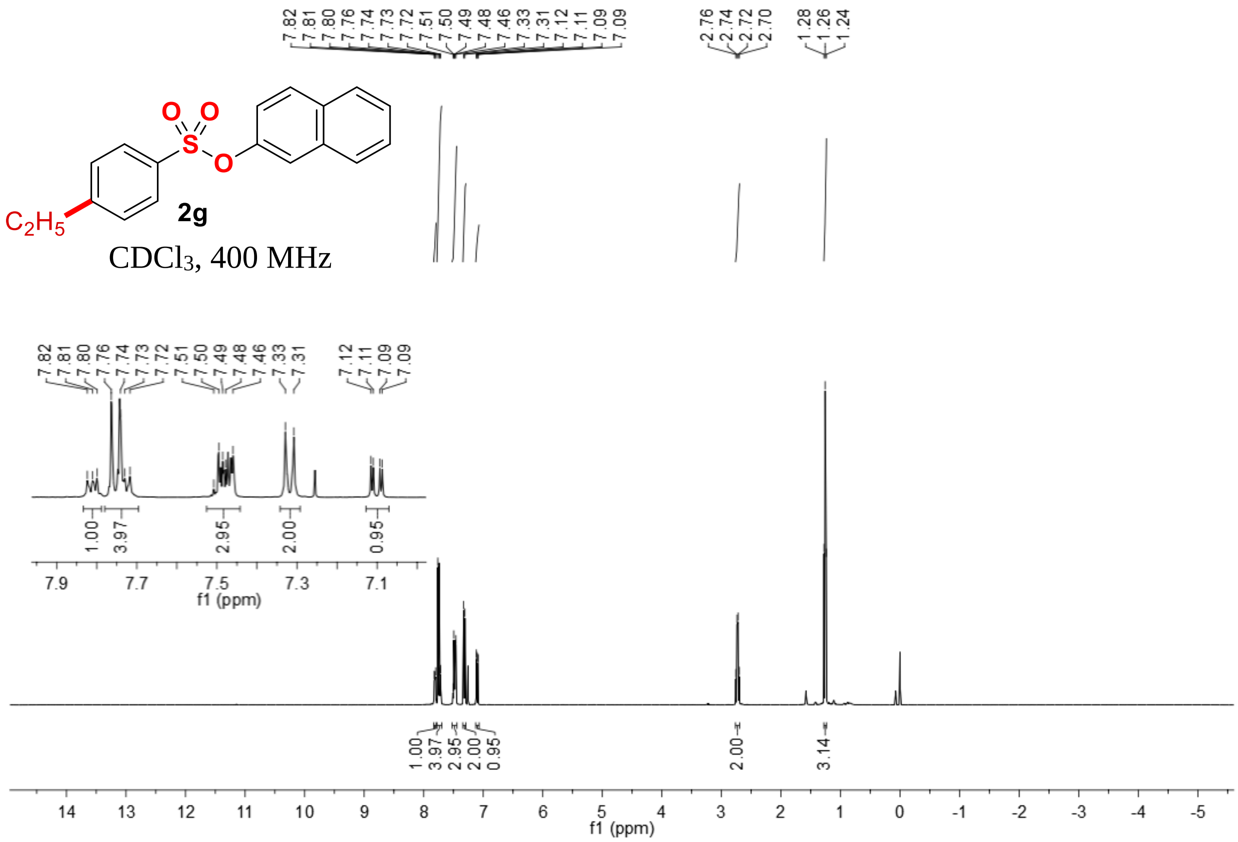
2g
CDCl3, 400 MHz
f1 (ppm)

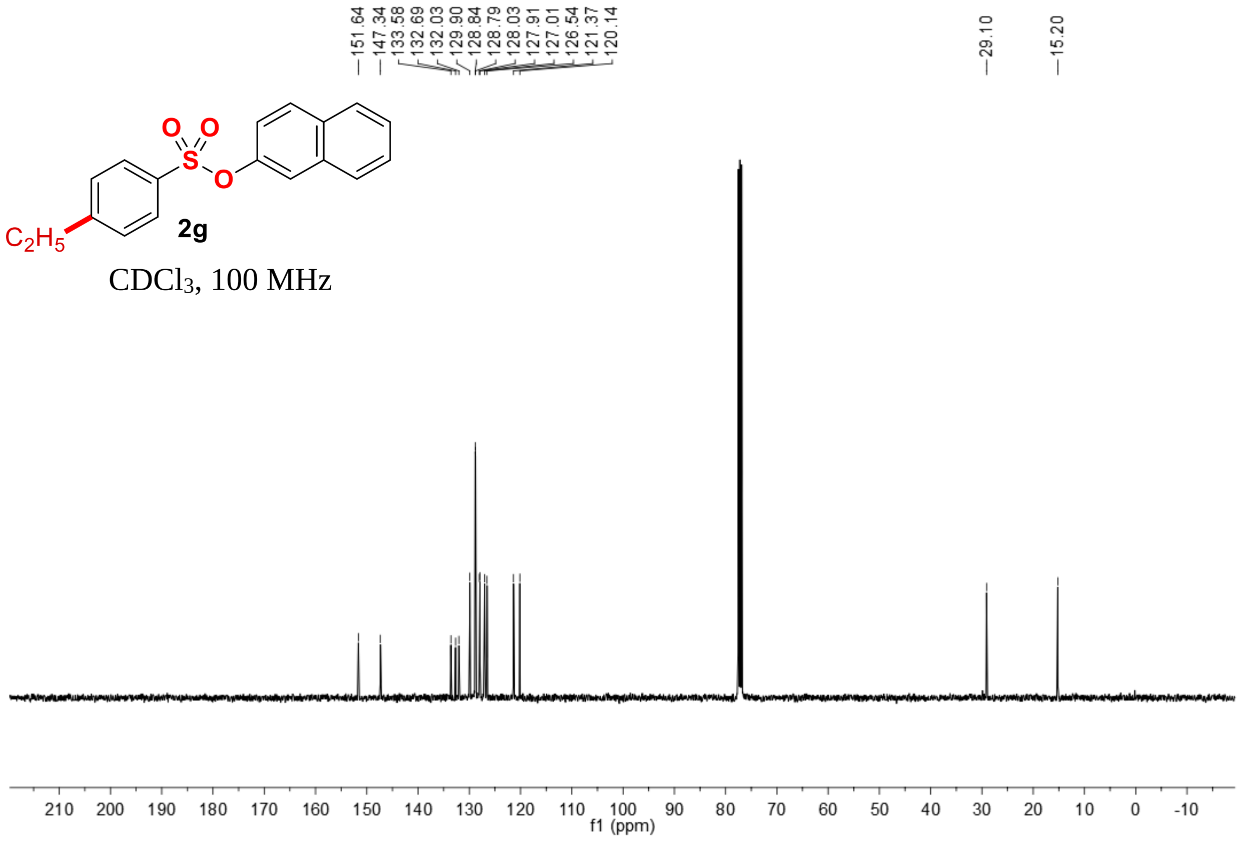
2g
CDCl3, 100 MHz
f1 (ppm)

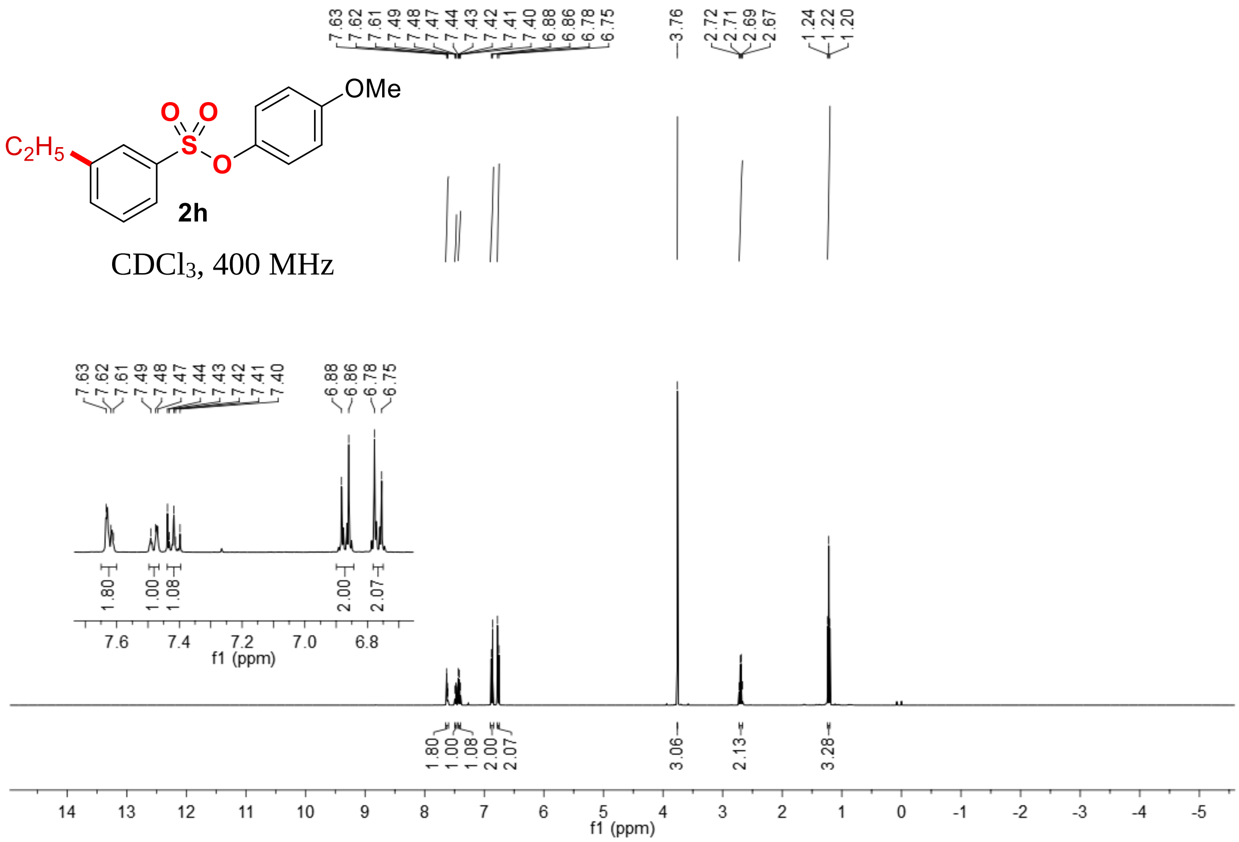
OMe
C2H5
2h
CDCl3, 400 MHz
f1 (ppm)

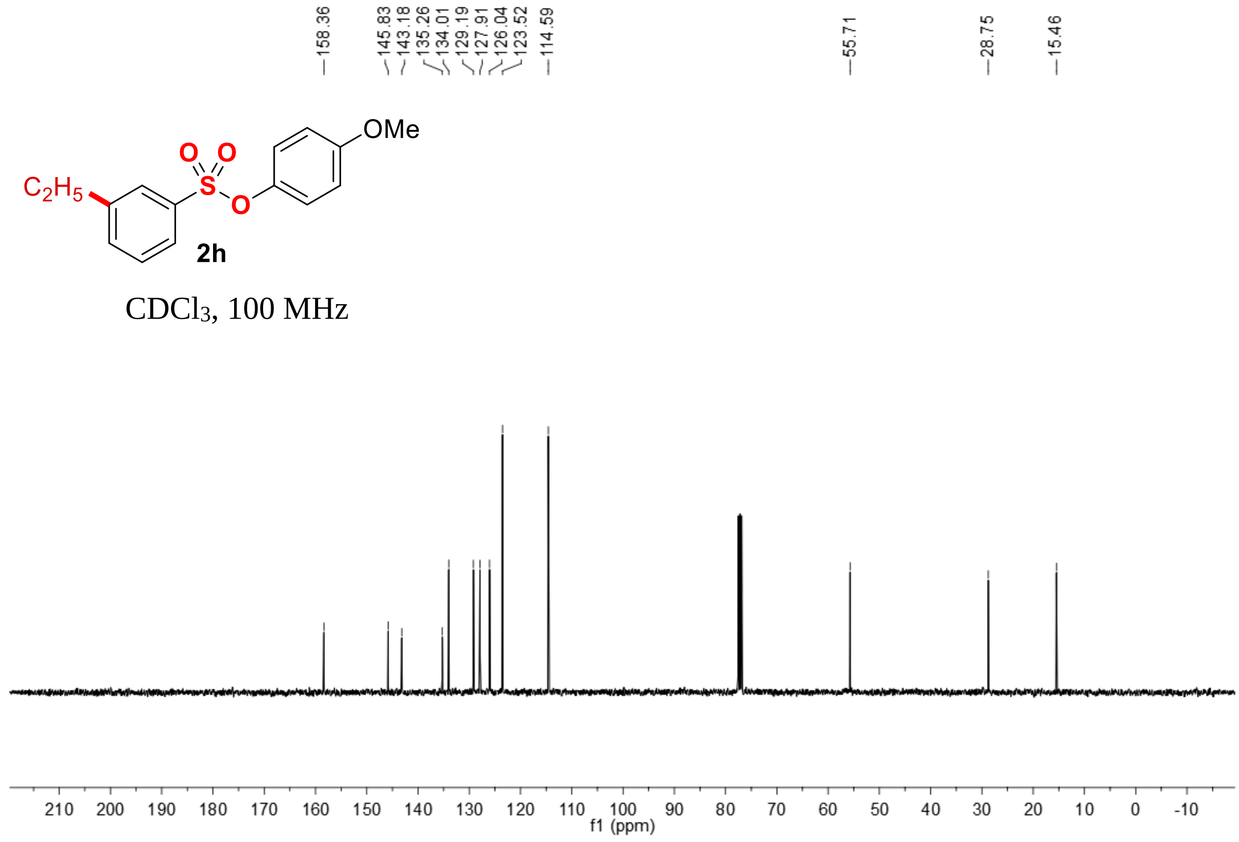
OMe
C2H5
2h
CDCl3, 100 MHz
f1 (ppm)

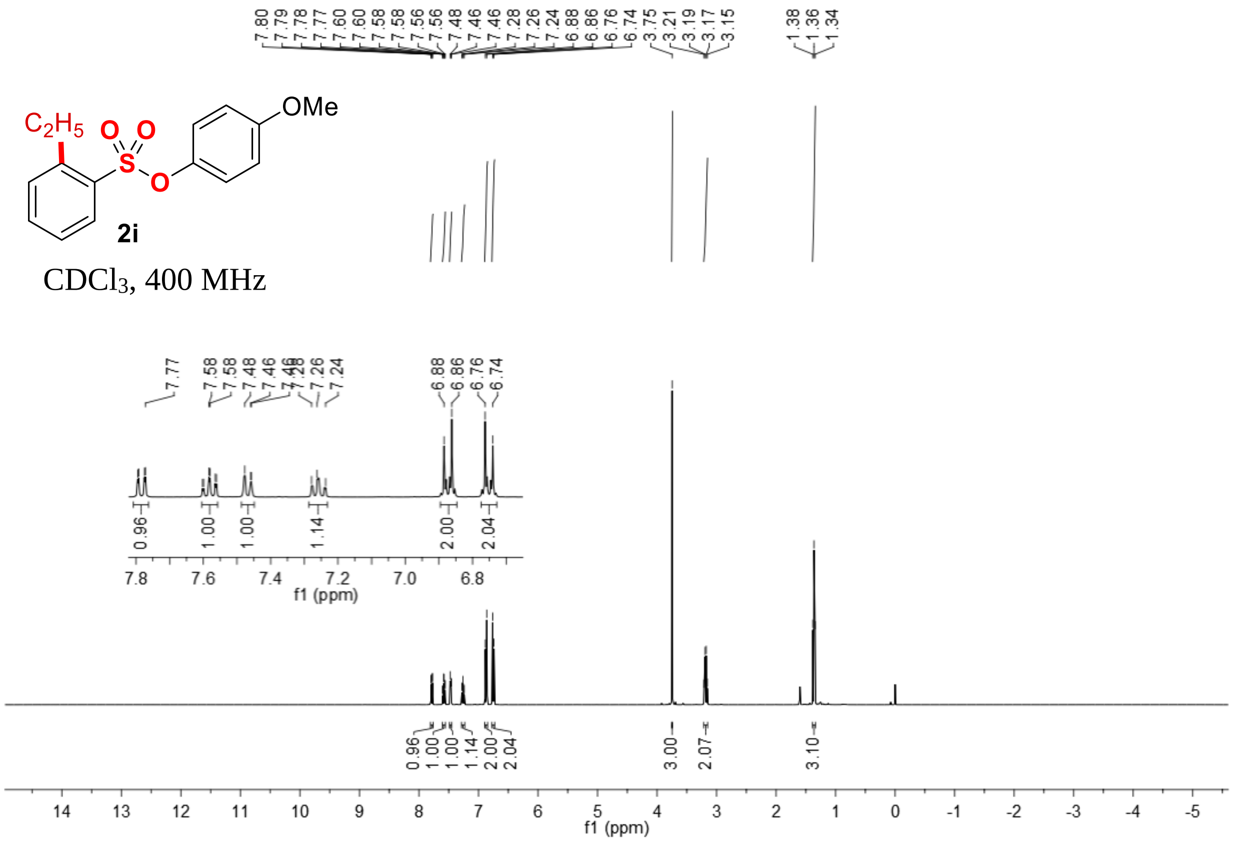

OMe
C_2H_5
2i
CDCl3, 400 MHz
f1 (ppm)

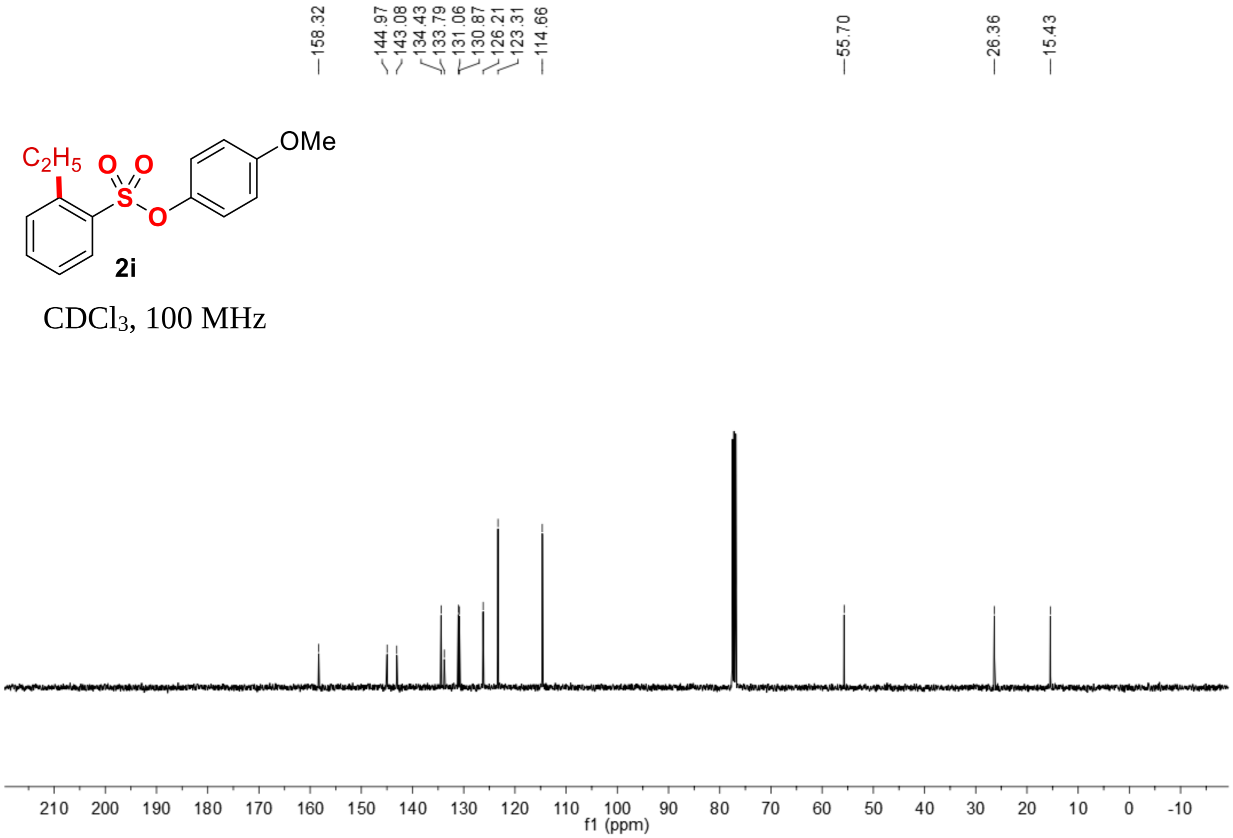

OMe
C_2H_5
2i
CDCl3, 100 MHz
f1 (ppm)

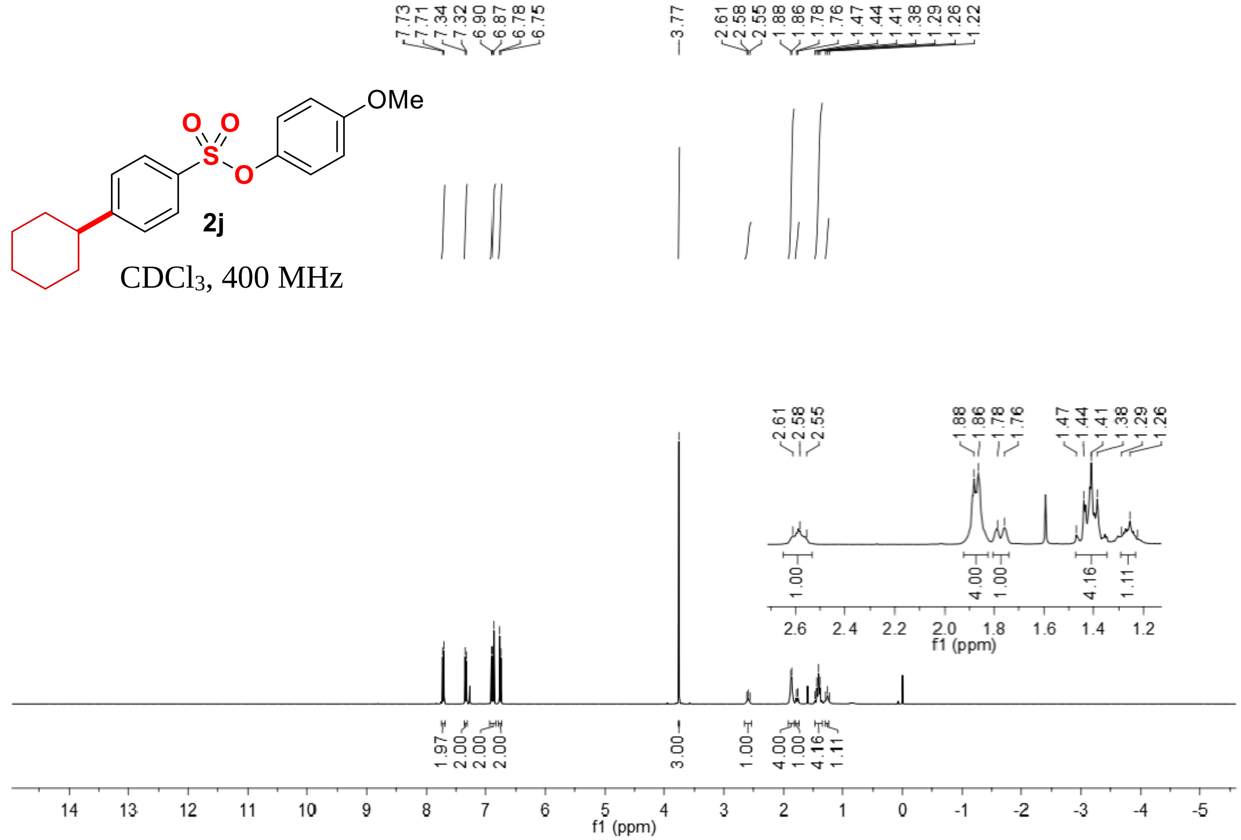
2j
CDCl3, 400 MHz
f1 (ppm)

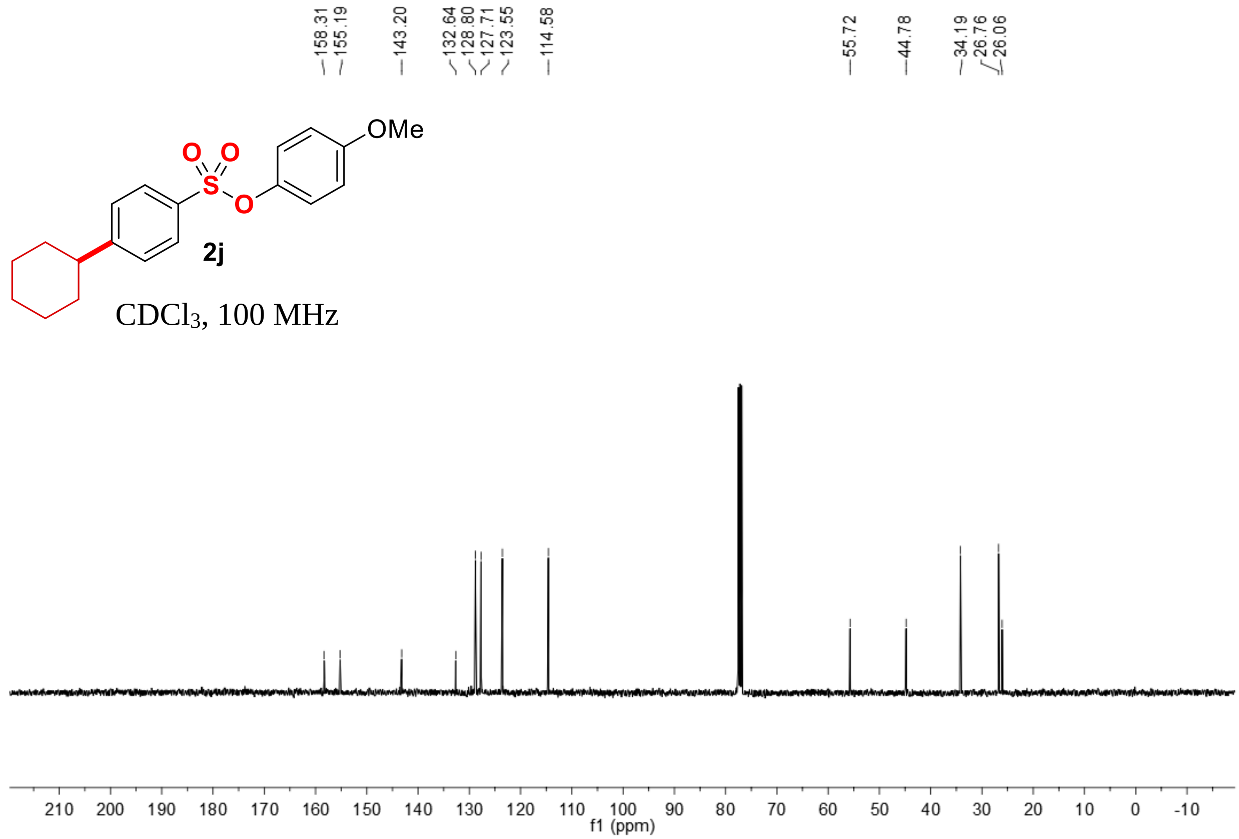
2j
CDCl3, 100 MHz
f1 (ppm)

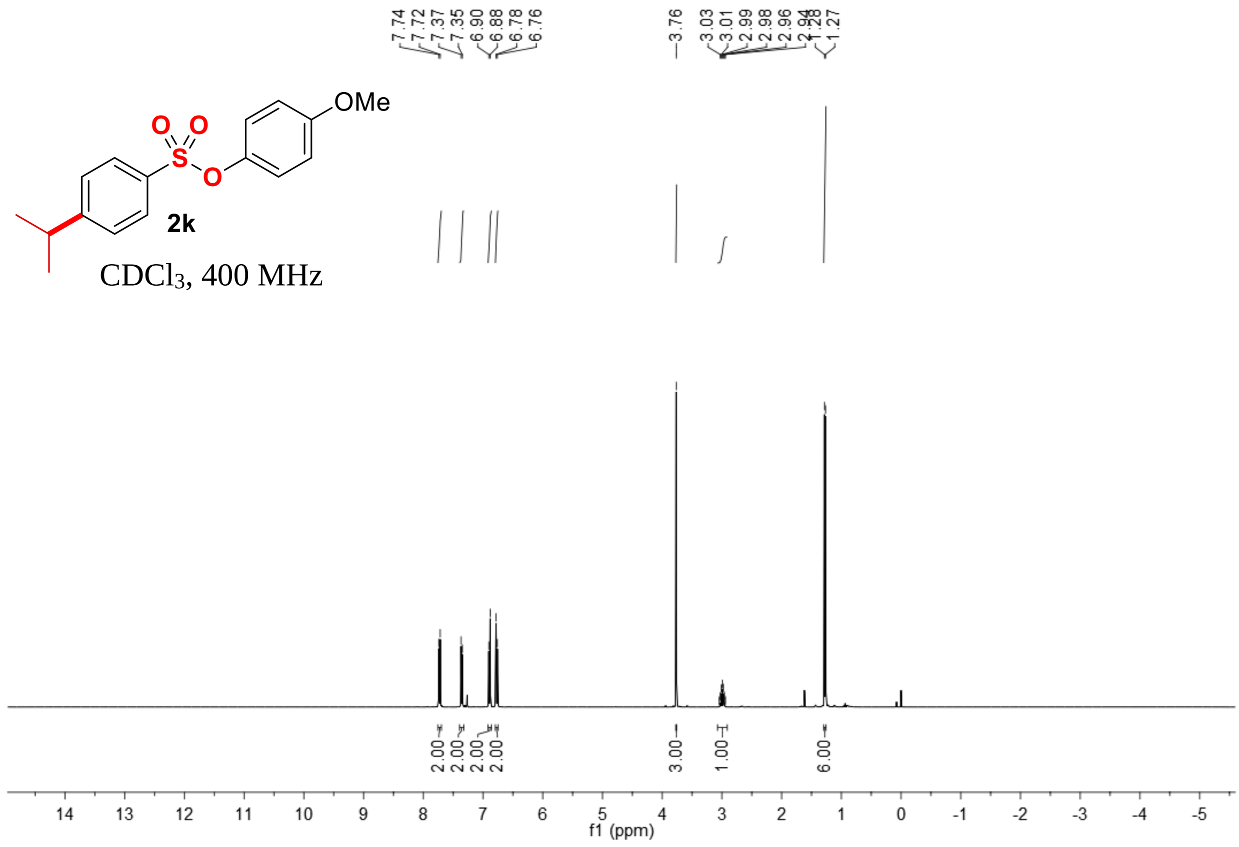
OMe
2k
CDCl3, 400 MHz
7.74
7.72
7.37
7.35
6.90
6.88
6.78
6.76
3.76
3.03
3.01
2.99
2.98
2.96
2.94
1.28
1.27
2.00
2.00
2.00
2.00
3.00
1.00
6.00
f1 (ppm)

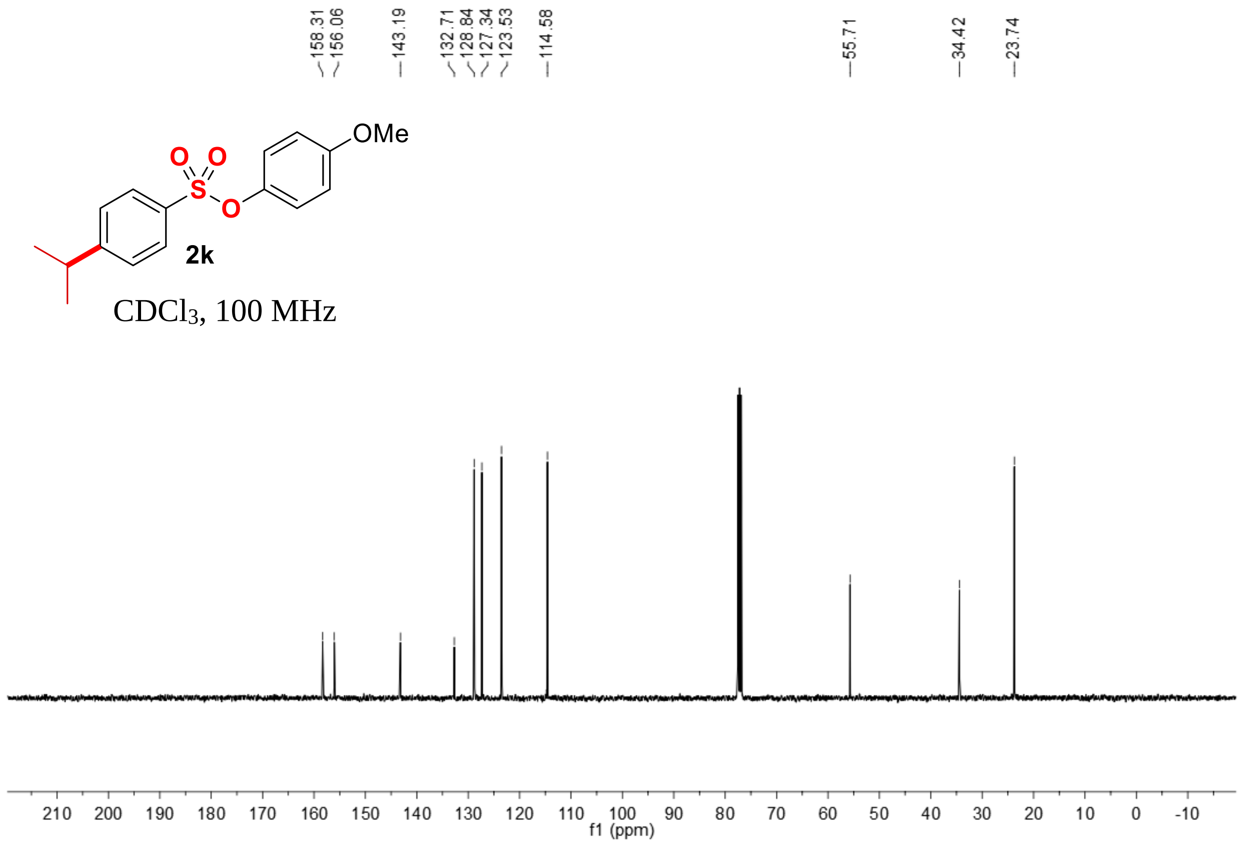
OMe
2k
CDCl3, 100 MHz
158.31
156.06
143.19
132.71
128.84
127.34
123.53
114.58
55.71
34.42
23.74
f1 (ppm)

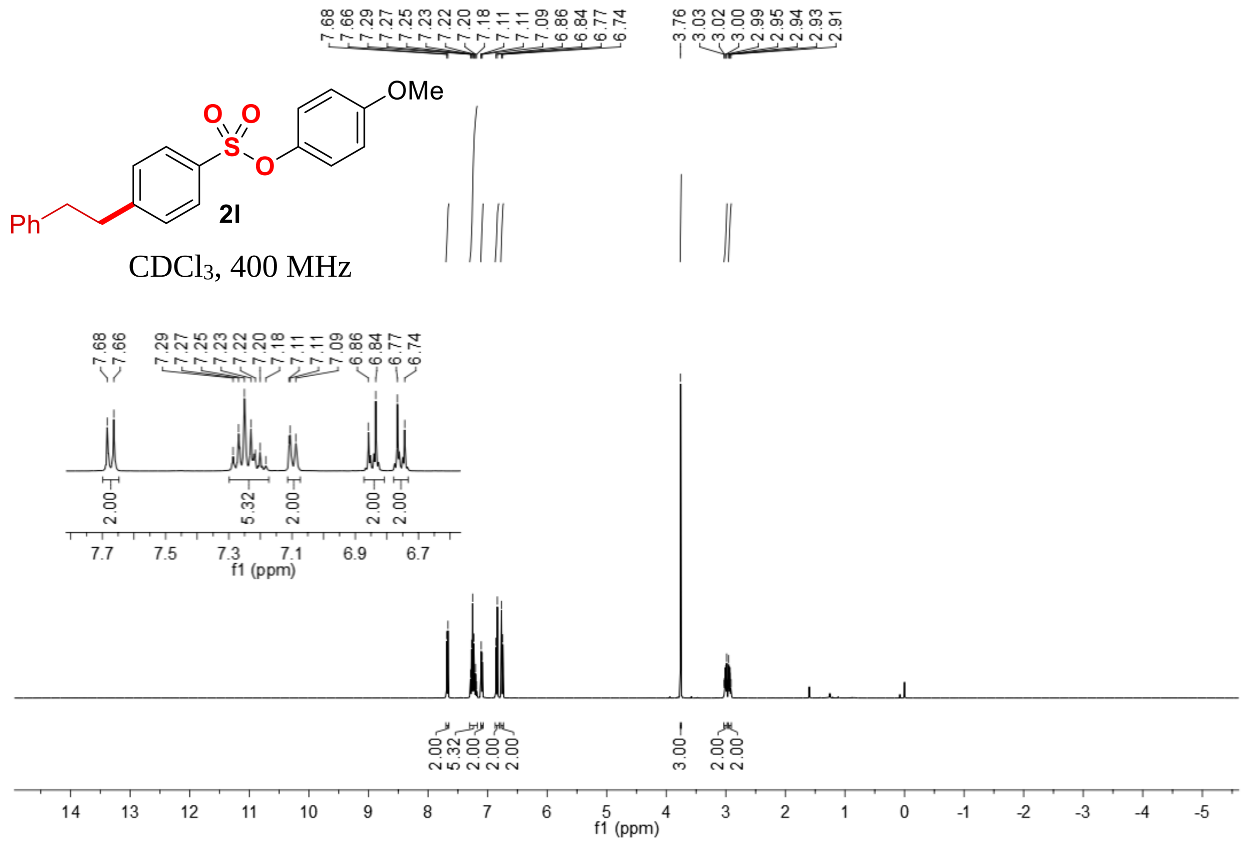
7.68
7.66
7.29
7.27
7.25
7.23
7.22
7.20
7.18
7.11
7.11
7.09
6.86
6.84
6.77
6.74
3.76
3.03
3.02
3.00
2.99
2.95
2.94
2.93
2.91
OMe
O
O
S
O
Ph
2l
CDCl3, 400 MHz
2.00
5.32
2.00
2.00
2.00
7.7
7.5
7.3
7.1
6.9
6.7
f1 (ppm)
3.00
14
13
12
11
10
9
8
7
6
5
4
3
2
1
0
-1
-2
-3
-4
-5

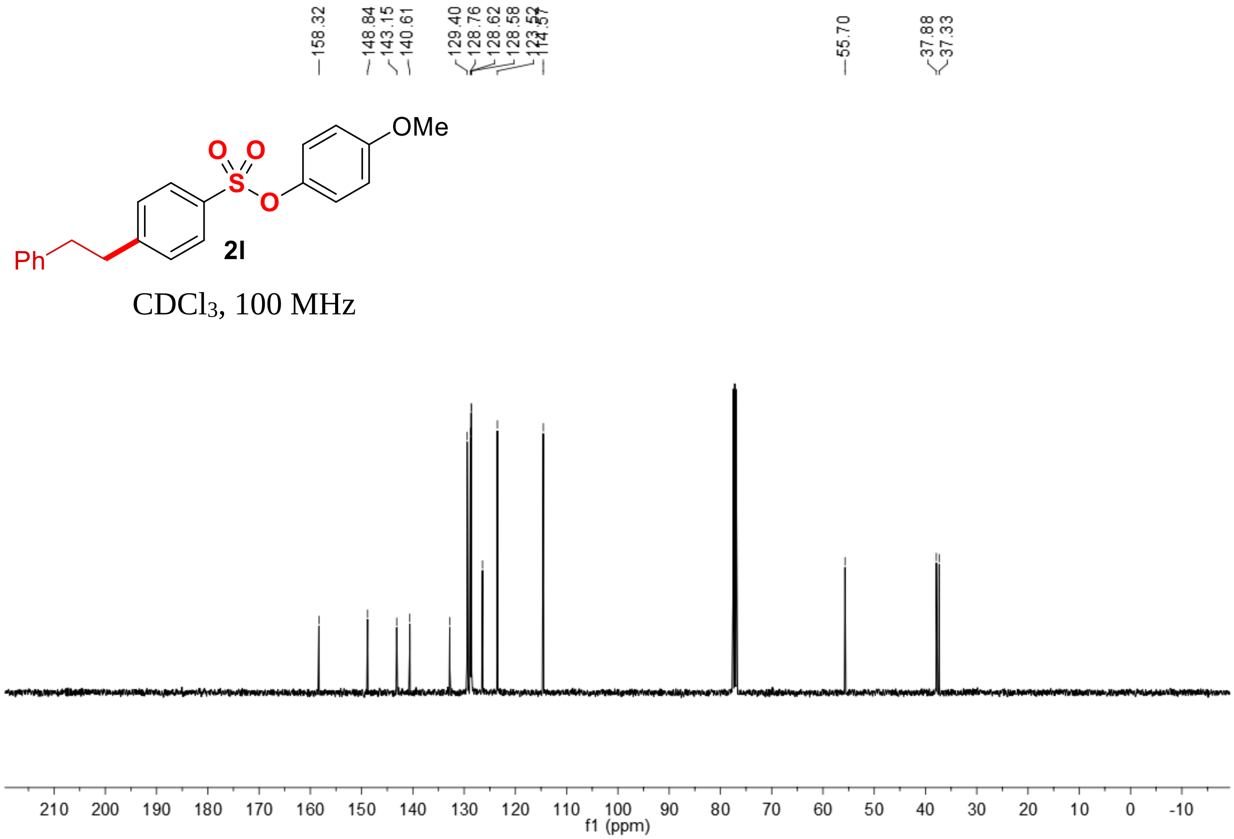
158.32
148.84
143.15
140.61
129.40
128.76
128.62
128.58
123.52
114.57
55.70
37.88
37.33
OMe
O
O
S
O
Ph
2l
CDCl3, 100 MHz
210
200
190
180
170
160
150
140
130
120
110
100
90
80
70
60
50
40
30
20
10
0
-10
f1 (ppm)

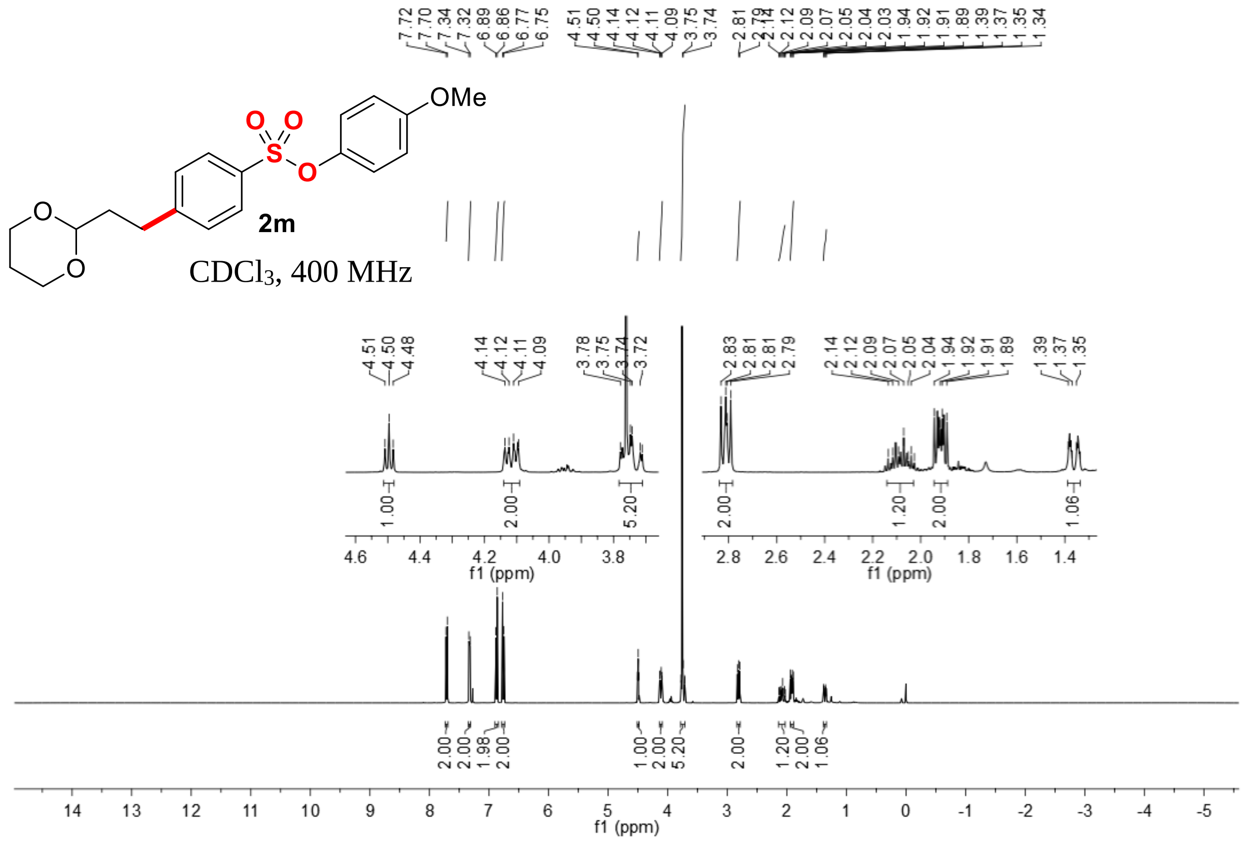
7.72
7.70
7.34
7.32
6.89
6.86
6.77
6.75
4.51
4.50
4.14
4.12
4.11
4.09
3.75
3.74
2.81
2.79
2.14
2.12
2.09
2.07
2.05
2.04
2.03
1.94
1.92
1.91
1.89
1.39
1.37
1.35
1.34
OMe
2m
CDCl3, 400 MHz
f1 (ppm)

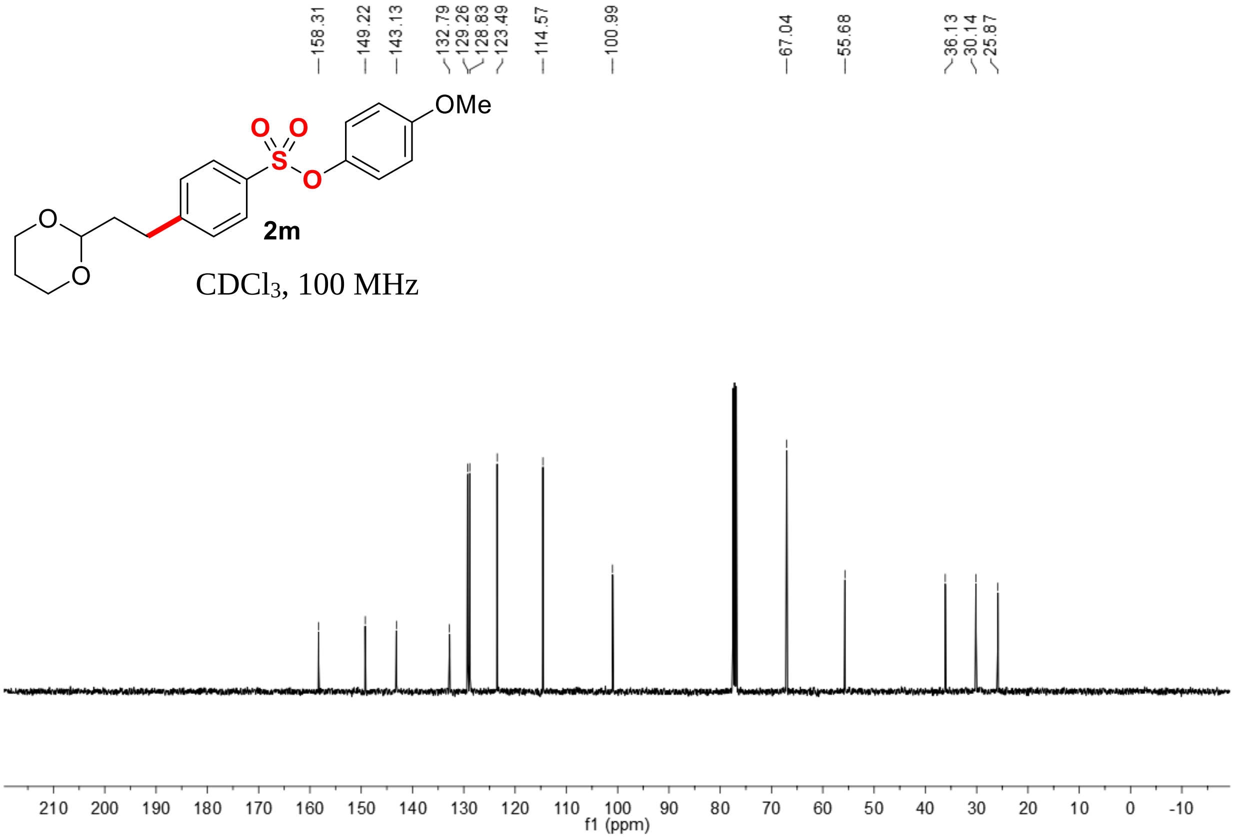
158.31
149.22
143.13
132.79
129.26
128.83
123.49
114.57
100.99
67.04
55.68
36.13
30.14
25.87
OMe
2m
CDCl3, 100 MHz
f1 (ppm)
